# Identifying potentially invasive non‐native marine and brackish water species for the Arabian Gulf and Sea of Oman

**DOI:** 10.1111/gcb.14964

**Published:** 2020-02-04

**Authors:** Stacey A. Clarke, Lorenzo Vilizzi, Laura Lee, Louisa E. Wood, Winston J. Cowie, John A. Burt, Rusyan J. E. Mamiit, Hassina Ali, Phil I. Davison, Gemma V. Fenwick, Rogan Harmer, Michał E. Skóra, Sebastian Kozic, Luke R. Aislabie, Adam Kennerley, Will J. F. Le Quesne, Gordon H. Copp, Paul D. Stebbing

**Affiliations:** ^1^ Centre for Environment, Fisheries and Aquaculture Science Lowestoft UK; ^2^ Department of Ecology and Vertebrate Zoology Faculty of Biology and Environmental Protection University of Łódź Łódź Poland; ^3^ Department of Evolution, Ecology and Behaviour Institute of Integrative Biology University of Liverpool Liverpool UK; ^4^ Centre for Environment, Fisheries and Aquaculture Science Weymouth UK; ^5^ Environment Agency – Abu Dhabi Abu Dhabi United Arab Emirates; ^6^ Centre for Genomics and Systems Biology New York University Abu Dhabi Abu Dhabi United Arab Emirates; ^7^ Global Green Growth Institute Abu Dhabi United Arab Emirates; ^8^ Ministry of Climate Change and Environment Dubai United Arab Emirates; ^9^ Lancaster Environment Centre Lancaster University Lancashire UK; ^10^ Faculty of Oceanography and Geography Institute of Oceanography University of Gdańsk Hel Poland; ^11^ Department of Life & Environmental Sciences Bournemouth University Poole UK; ^12^ Environmental & Life Sciences Graduate Program Trent University Peterborough Canada; ^13^ Present address: APEM Ltd A17 Embankment Business Park Heaton Mersey Manchester SK4 3GN UK

**Keywords:** AS‐ISK, extant non‐native species, horizon species, risk screening, ROPME

## Abstract

Invasive non‐native species (NNS) are internationally recognized as posing a serious threat to global biodiversity, economies and human health. The identification of invasive NNS is already established, those that may arrive in the future, their vectors and pathways of introduction and spread, and hotspots of invasion are important for a targeted approach to managing introductions and impacts at local, regional and global scales. The aim of this study was to identify which marine and brackish NNS are already present in marine systems of the northeastern Arabia area (Arabian Gulf and Sea of Oman) and of these which ones are potentially invasive, and which species have a high likelihood of being introduced in the future and negatively affect biodiversity. Overall, 136 NNS were identified, of which 56 are already present in the region and a further 80 were identified as likely to arrive in the future, including fish, tunicates, invertebrates, plants and protists. The Aquatic Species Invasiveness Screening Kit (AS‐ISK) was used to identify the risk of NNS being (or becoming) invasive within the region. Based on the AS‐ISK basic risk assessment (BRA) thresholds, 36 extant and 37 horizon species (53.7% of all species) were identified as high risk. When the impact of climate change on the overall assessment was considered, the combined risk score (BRA+CCA) increased for 38.2% of all species, suggesting higher risk under warmer conditions, including the highest‐risk horizon NNS the green crab *Carcinus maenas*, and the extant macro‐alga *Hypnea musciformis*. This is the first horizon‐scanning exercise for NNS in the region, thus providing a vital baseline for future management. The outcome of this study is the prioritization of NNS to inform decision‐making for the targeted monitoring and management in the region to prevent new bio‐invasions and to control existing species, including their potential for spread.

## Graphical abstract

This review identifies and risk screens potentially invasive non‐native species (NNS) within the Regional Organization for the Protection of the Marine Environment Sea Area. In total, 136 NNS were identified, of which 56 are already present and a further 80 were identified as likely to arrive in the future.

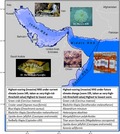

## 1 INTRODUCTION

The increasing degradation of marine and brackish habitats around the globe is drawing attention to the importance of protecting these environments, especially from human‐mediated impact. This is especially true for the Arabian Gulf and the Sea of Oman, a region that falls within the area of the Regional Organization for the Protection of the Marine Environment (ROPME), which has the mandate for supporting cooperative management of the ROPME Sea Area (RSA; Bailey & Munawar, 2015; Van Lavieren & Klaus, 2013). The RSA, which is bordered by the countries of Bahrain, Iran, Iraq, Kuwait, Oman, Qatar, Saudi Arabia and the United Arab Emirates, has unique environmental features, including a marine environment characterized by extreme oceanographic and meteorological conditions (Riefl et al., 2012; Sale et al., 2011; Van Lavieren et al., 2011; Vaughan, Al‐Mansoori, & Burt, 2019). Sea surface temperatures (SST) in the RSA regularly exceed 37°C during the extreme summer months (Paparella, Xu, Vaughan, & Burt, 2019), and mean salinity is 42 ppt, with >50 ppt common in the south and up to 70 ppt in coastal lagoons (Vaughan et al., 2019; Wabnitz et al., 2018).

Characterized by low species diversity, with many species already living at the margins of survival, the RSA is particularly sensitive to human‐generated impacts (Sheppard et al., 2010; Vaughan et al., 2019), which are exacerbated by a rapidly increasing human population and increased use of the marine environment (Bailey & Munawar, 2015; Burt, 2014; Burt, Al‐Harthi, & Al‐Cibahy, 2011; Riefl et al., 2012; Sale et al., 2011; Sheppard et al., 2010; United Nations, 2017; Van Lavieren et al., 2011; Van Lavieren & Klaus, 2013). Particularly detrimental is the rise in temperature and salinity (IPCC, 2007), and the large decrease in input of fresh water from the River Shat Al Arab, which has increased salinity at the northern end of the RSA (UN‐ESCWA & BGR, 2013). These are aggravated by extensive use of sea water as a coolant for power stations or directly for desalination—processes that release warmer, more saline water back into the sea (AGEDI, 2016; Elimelech & Phillip, 2011; Jenkins, Paduan, Roberts, Schlenk, & Weis, 2012). The original area of coral reef cover in the RSA has declined by 70%, with most of the remainder either threatened or in a process of severe degradation (Vaughan et al., 2019; Wilkinson, 2008).

It is commonly recognized that invasive non‐native species (NNS) are one of the greatest threats to global biodiversity and are a key driver in ecosystem change, especially when introduced into sensitive environments (Costello et al., 2010; Kideys, 2002; Rockström et al., 2009). The International Maritime Organization (IMO) recognizes the introduction of harmful aquatic organisms (including NNS and pathogens) to new environments as one of the four greatest threats to the world's oceans—the other three being land‐sourced marine pollution, overexploitation of living marine resources and destruction of habitat (IMO, 2018). In recognition of this increasing threat and to manage more effectively the risks posed by invasive NNS, the Convention on Biological Diversity (CBD) has set international targets and frameworks for global action. Specifically, the CBD has provided Guiding Principles for the Prevention, Introduction and Mitigation of Impacts of Alien Species that Threaten Ecosystems, Habitats and Species (United Nations, 2002). These guidelines include a three‐stage hierarchical approach based on (a) prevention of introduction; (b) early detection and rapid action (e.g. eradication, where feasible) in the event of a new introduction to prevent establishment; and (c) where eradication is not feasible, control and containment measures.

Particularly important actions include the identification of potential invasive NNS that could enter a region, early detection of those already there (Chan et al., 2019; Ojaveer et al., 2018), and prevention of further introductions. On the contrary, post‐introduction actions such as eradication, control and containment are generally difficult and unlikely to be successful (Williams & Grosholz, 2008), particularly in the marine environment (Werschkun et al., 2014).

Understanding the main vectors and pathways of introduction and spread into, and within, a region enables targeted NNS management by identifying the locations most at risk from introductions (Tidbury, Taylor, Copp, Garnacho, & Stebbing, 2016). Within the RSA, key potential vectors of introduction of NNS include ship traffic of which there are the large volumes entering the area from international ports (Sale et al., 2011; Vaughan et al., 2019), especially from India, China and Pakistan (Automatic Identification System [AIS] data obtained on request from Marine Traffic: http://www.marinetraffic.com/en/p/ais-historical-data). Other introduction vectors include recreational boating, cruise ships and aquaculture, and, to a lesser extent, the aquarium trade (Miza, Majiedt, & Sink, 2014). The key vectors involved in marine introduction vectors are ballast water discharge, hull fouling, general fouling, hitchhiking and release (intentional or accidental: Minchin, Gollasch, Cohen, Hewitt, & Olenin, 2009). In terms of aquaculture, this industry is increasing with more than $15 billion worth of projects being planned in the RSA for the current decade (Innovation Norway, 2015), and due to limited freshwater resources, most countries in the RSA are actively researching future options for farming marine species.

**Figure 1:**
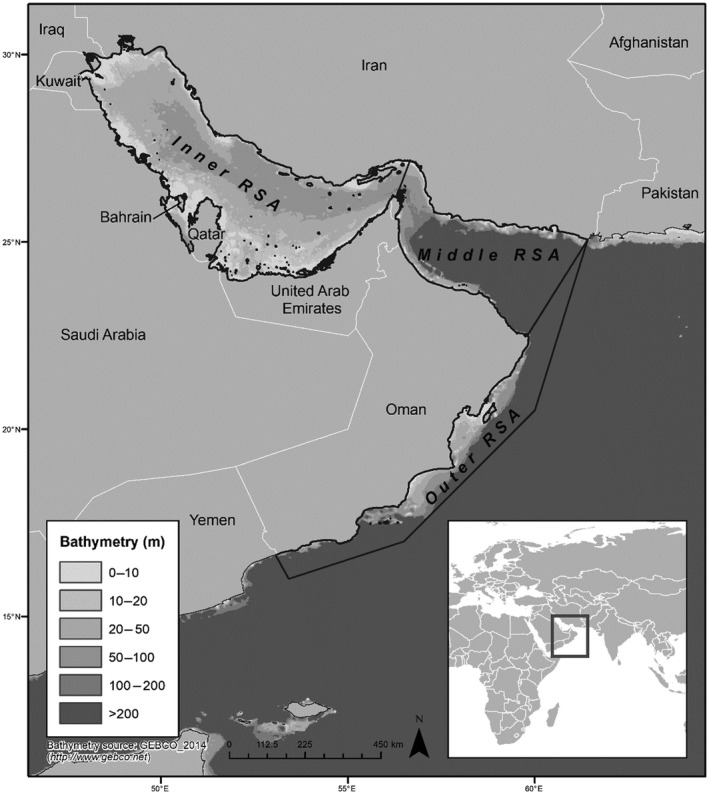
Map of the study (risk assessment) area, the Regional Organization for Protection of the Marine Environment (ROPME) Sea Area showing the Inner and Middle Sea Areas which were the focus of this study

The present study identifies potentially invasive marine and brackish water NNS in the Arabian Gulf and Sea of Oman. This area also coincides with the Inner and Middle RSA and is referred to hereafter as the risk assessment area (Figure 1). The specific objectives are to (a) identify extant NNS in the risk assessment area; (b) complete a horizon‐scanning exercise to determine which marine and brackish NNS are likely to arrive in the risk assessment area in the foreseeable future; (c) complete risk screenings of both sets of (extant and horizon) species using a widely tested electronic decision‐support toolkit with regard to current and future climate conditions; (d) calibrate and validate the resulting dataset, and therefore classify the NNS as being of low‐to‐medium and high risk of being (or becoming) invasive in the risk assessment area and (e) evaluate the confidence level (CL) of the assessments. Notably, this is the first horizon‐scanning and risk‐identification exercise for marine and brackish NNS for the RSA, and the outcomes are intended to provide decision‐makers with evidence upon which to develop informed policy and prioritized management strategies for protection of the area's unique marine and brackish water environments from adverse impacts of NNS.

## 2 MATERIALS AND METHODS

### 2.1 Risk screening

The Aquatic Species Invasiveness Screening Kit (AS‐ISK), which is available for free download at http://www.cefas.co.uk/nns/tools, was used to identify potentially invasive NNS with respect to the risk assessment area. Described in detail in Copp et al. (2016), the AS‐ISK is fully compliant with the ‘minimum standards’ (Roy et al., 2018) for the assessment of NNS for the European Commission Regulation on the prevention and management of the introduction and spread of invasive alien species (European Commission, 2014). The AS‐ISK consists of 55 questions: the first 49 questions cover the biogeography/historical and biology/ecology aspects of the species under assessment, including risks of introduction, establishment, dispersal and impact, and comprise the basic risk assessment (BRA). The other six questions require the assessor to predict how future climatic conditions are likely to affect the BRA with respect to risks of introduction, establishment, dispersal and impact, and these comprise the climate change assessment (CCA). In the recently released AS‐ISK v2, which the assessors employed in the current study, the 16 taxonomic groups of aquatic organisms previously accounted for in AS‐ISK v1 (Copp et al., 2016) have been expanded to a total of 27 following the classification of living organisms by Ruggiero et al. (2015).

For each question in AS‐ISK, the assessor must provide a response, justification and level of confidence, and the screened species eventually receives both a BRA and a BRA+CCA (composite) score (respectively, ranging from −20.0 to 68.0 and from −32.0 to 80.0). AS‐ISK scores <1.0 suggest that the species is unlikely to become invasive in the risk assessment area and is therefore classified as ‘low risk’. Higher scores classify the species as posing either a ‘medium risk’ or a ‘high risk’ of becoming invasive. Distinction between medium‐ and high‐risk levels depends upon setting a ‘threshold’ value, which is typically obtained through risk assessment area‐specific ‘calibration’ subject to availability of a representative sample size (i.e. number of screened species), which was recently estimated at *n* = 15–20 (Vilizzi et al., 2019). For the purposes of this study, with regard to the CCA component of the screening process, current predictions for the RSA suggest an increase in SST between 0.5 and 1.4°C and salinity increases of up to 18 ppt by 2050 (Vaughan et al., 2019; Wabnitz et al., 2018). The assessors used this scenario to provide a consistent outlook on the provision of CCA scoring.

The ranked levels of confidence (1 = low; 2 = medium; 3 = high; 4 = very high) associated with each response in AS‐ISK mirror the confidence rankings that the Intergovernmental Panel on Climate Change recommended (IPCC, 2005; see also Copp et al., 2016). Based on the CL allocated to each response for a given species, the confidence factor (CF) is calculated as:

$$\sum \left({\text{CL}}_{Qi}\right)/\left(4\times 55\right)\;\left(i=1,\;\ldots ,\;55\right),$$

where CL_Q_ *_i_* is the confidence level for Question *i* (Q*i*), 4 is the maximum achievable value for certainty of confidence in the response (i.e. ‘very high’) and 55 is the total number of questions comprising the AS‐ISK questionnaire. The CF ranges from a minimum of 0.25 (i.e. all 55 questions with CL equal to 1) to a maximum of 1 (i.e*.* all 55 questions with CL equal to 4). Two additional CFs were also computed, namely the CF_BRA_ and the CF_CCA_ based on the 49 questions in the BRA and the six questions in the CCA, respectively.

### 2.2 NNS selection

#### 2.2.1 Extant

The initial list of NNS recorded in region thus far was compiled using a variety of relevant search terms in Google and Google Scholar, personal bibliographic collections, and NNS databases including the Global Invasive Species Database (GISD; http://www.iucngisd.org/gisd/), Invasive Species Compendium (CABI; http://www.cabi.org/isc) and Global Register of Introduced and Invasive Species (Griis; http://www.griis.org/). These were employed to summarize the existing knowledge of marine and brackish water organisms that are known or suspected to be non‐native to any of the countries in the risk assessment area. In‐region experts reviewed and validated the initial list through various consultations. For each species identified as potentially being a NNS present, additional information was gathered including (a) taxonomy; (b) habitat; (c) whether the organism has been recorded or suspected to be in the risk assessment area; (d) whether it is acknowledged to be introduced, established or spreading; (e) the known and potential impacts it may have; (f) the introduction vector and potential pathway as per CBD groupings, that is, intentional release, including biological control, and other releases; escape, including aquaculture, aquarium trade; transport (stowaway), including ballast water, hull fouling, and other transport); (g) the specific location where it was reported and (h) the date it was first recorded. Cryptogenic species (i.e. native/non‐native status in the risk assessment area uncertain), or those for which the basis of identification in the risk assessment area was derived from limited records, remained on the list unless expert judgement indicated otherwise. To reduce risk of double‐counting the same species under different names, the World Register of Marine Species (http://www.marinespecies.org/) was used to determine the current and previously accepted genus and species names. Where sources varied in their conclusion of invasiveness of a species in the risk assessment area, the most recent scientific manuscripts were used where available (alongside in‐region expert knowledge) to determine the decision to add or not to the list.

#### 2.2.2 Horizon

The assessors generated the horizon list using (a) a combination of literature searches; (b) predictions by the CABI Horizon‐Scanning tool (http://www.cabi.org/HorizonScanningTool); (c) refinement of in‐region lists where more detailed information obtained during the screening process clarified that the species was not yet present in the risk assessment area (i.e. it may be present in Iran, but in the Caspian Sea rather than in the Inner or Middle RSA) and (d) a review of aquaculture in the Inner and Middle RSA (i.e. those NNS being used by the industry or being reviewed for future use, but not yet recorded as present outside of cultivation). For the CABI tool, the following search criteria were used: (a) recipient countries selected: only those in‐region; (b) source countries selected: neighbouring countries and other countries with matching climate type listed; (c) vectors selected: all, with the exception of those that were considered not applicable to marine species (i.e. Containers & packaging; Machinery & equipment; Mulch, straw, baskets & sod; Soil, sand & gravel; Germplasm; Hides, trophies & feathers; Wind dispersal) and (d) habitats selected: brackish and marine. ‘Brackish’ was included in the search terms as there is potential for brackish water species to survive in the risk assessment area if they have a marine stage to their life cycle and/or a broad salinity tolerance that enables them to survive in marine habitats. The initial list was then manually reviewed and validated, especially in relation to climate suitability. Despite the climate matching criteria in CABI being selected to restrict to similar climate types, there were some species that were evidently not suited to waters of the temperatures found in the risk assessment area. These were removed from the list unless there was evidence of the species being established in similarly harsh environments elsewhere.

### 2.3 Data processing

Following computation of the BRA and BRA+CCA scores with AS‐ISK, receiver operating characteristic (ROC) curve analysis (Bewick, Cheek, & Ball, 2004) was used to assess the predictive ability of AS‐ISK to discriminate between species posing a high risk and those posing a medium or low risk of being invasive for the risk assessment area. The implementation of the ROC curve analysis requires a priori categorization in terms of documented invasiveness (i.e. non‐invasive or invasive) of species. However, unlike fishes and lampreys, for which a priori categorization is facilitated by the availability of online databases providing all required information (i.e. FishBase; http://www.fishbase.org; cf. Bilge, Filiz, Yapici, Tarkan & Vilizzi, 2019; Glamuzina et al., 2017; Li, Chen, Wang, & Copp, 2017; Tarkan, Sarı, İlhan, Kurtul, & Vilizzi, 2017; Tarkan, Vilizzi, et al., 2017; Zięba, Vilizzi, & Copp, 2020), this study adopted an ‘integrated approach’ to determine the a priori invasiveness status of species in all other aquatic organism groups (other than freshwater and marine fishes and lampreys, as identified in AS‐ISK) due to the more limited information available.

The integrated approach followed four steps: (a) similar to fishes and lampreys (cf. FishBase), there was a preliminary consultation of SeaLifeBase (http://www.sealifebase.org) for any reference to the species' threat to humans, with the species categorized a priori as invasive if listed as ‘potential pest’ and as non‐invasive if listed as ‘harmless’; (b) in case the species was listed as either ‘not evaluated’ or was absent in the above database, then a search was made of the GISD (http://www.iucngisd.org/gisd/), with the species categorized a priori as invasive if listed therein; (c) in case the species was absent from the GISD, then an additional search was made of the continent‐level lists for invasive species in Africa, Asia, Europe, North America, South America and Australia, whereby the species was categorized a priori as ‘invasive’ if it appeared in the generated list and finally; (d) in case the species was absent from any of the previous databases, then a Google Scholar (literature) search was performed (using the keywords ‘invasive’, ‘invasiveness’ and ‘impact’ along with that of the species) to check whether at least one peer‐reviewed reference in support was found. The latter was then taken as ‘sufficient evidence’ for categorizing the species a priori as invasive; whereas, if no evidence was found, then the species was categorized a priori as non‐invasive. Notably, in case a species was listed as harmless in FishBase or SeaLifeBase but found to be invasive in any of the other steps of the process, then the a priori categorization of the species became that of invasive.

### 2.4 Statistical analysis

A ROC curve is a graph of sensitivity versus 1—specificity (or alternatively, sensitivity vs. specificity) for each threshold value, where in the present context sensitivity and specificity will be the proportion of a priori invasive and non‐invasive species, respectively, for the risk assessment area that AS‐ISK correctly identified as such. A measure of the accuracy of the calibration analysis is the area under the curve (AUC), which typically ranges from 0.5 to 1, and the closer to 1 the better the ability to differentiate between invasive and non‐invasive species. If the AUC is equal to 1, then the test is 100% accurate because both sensitivity and specificity are 1, and there are neither ‘false positives’ (a priori non‐invasive species classified as high risk, hence false invasive) nor ‘false negatives’ (a priori invasive species classified as low risk, hence false non‐invasive). Conversely, if the AUC is equal to 0.5, then the test is 0% accurate as it cannot discriminate between ‘true positives’ (a priori invasive species classified as high risk, hence true invasive) and ‘true negatives’ (a priori non‐invasive species classified as low risk, hence true non‐invasive). Following ROC analysis, the Youden's *J* statistic best determines the AS‐ISK threshold value that maximizes the true positives rate and minimizes the false‐positives rate, whereas a ‘default’ threshold of 1 was set to distinguish between low‐risk and medium‐risk species (see Section 2.1).

Receiver operating characteristic curve analysis was carried out with package pROC (Robin et al., 2011) for R x64 v3.2.0 (R Core Team, 2015) using 2,000 bootstrap replicates for the confidence intervals of specificities, which were computed along the entire range of sensitivity points (i.e. 0–1, at 0.1 intervals). For those groups of aquatic organisms for which a ‘representative’ sample size was available (i.e. *n* > 10), the aquatic organism‐specific thresholds could be estimated. However, in case of resulting mean AUC values <0.5, the corresponding threshold was discarded and the one for the ‘nearest’ aquatic organism combined group was used. The latter criterion applied also to any group including less than 10 screened species and for which ROC curve analysis was not possible.

Differences between mean CL_BRA_ and CL_CCA_ (see Section 2.1) depending upon species status (i.e. extant or horizon) were tested by permutational (univariate) analysis of variance (PERANOVA) based on a two‐factor design (i.e. factor Component, with the two levels BRA and CCA; factor Status, with the two levels Extant and Horizon), with both factors fixed (note that differences between mean CF_BRA_ and CF_CCA_ would lead the same outcomes being the two indices related). The analysis was carried out using PERMANOVA+ for PRIMER v6, with normalization of the data and using a Bray–Curtis dissimilarity measure, 9,999 unrestricted permutations of the raw data (Anderson, Gorley, & Clarke, 2008), and with statistical effects evaluated at *α* = 0.05 including a posteriori pairwise comparisons.

## 3 RESULTS

### 3.1 NNS selection

#### 3.1.1 Extant

The final list (Table S1) comprised 56 species from across Chromista (14; 25% of total), Arthropoda (10; 18%), Teleostei (10; 18%), Ascidiacea (seven; 13%), Plantae (five; 9%), Mollusca (four; 7%), Bryozoa (three; 5%) and Cnidaria (three; 5%). In total, 35 (63%) of these species were determined to be introduced, with the remaining 21 (38%) being cryptogenic. Native ranges of the 35 species recognized as NNS varied, with 12 (23%) coming from the Atlantic, seven (13%) from Southeast Asia, six (11%) from African waters, three (6%) from the Pacific, and another three (6%) from the Indian Ocean, and with the remaining species from a variety of smaller sea regions including the Mediterranean and Caspian seas. The most common suspected vector of introduction (as identified via expert knowledge based on species’ characteristics combined with information gathered from literature searches during the risk screening process) was via ballast water (36 instances, 51%), followed by fouling of equipment, vessel hulls or other hard surfaces (18, 26%), and aquaculture (10, 14%). The aquarium trade and mosquito (biological) control introduction vectors made up the remaining 9% (six instances). It is sometimes difficult to attribute introductions to specific vectors, and therefore the association of vectors to specific species introductions remains speculative. Several species had multiple vectors attributed to their introduction; hence, the numbers given here add up to more than the total number of species.

#### 3.1.2 Horizon scanning

The final list (Table S2) comprised 80 species from across Teleostei (22; 28% of total), Arthropoda (14; 18%), Mollusca (14; 18%), Plantae (seven, 9%), Annelida (five; 6%), Ascidiacea (five; 6%), Cnidaria (five; 6%), Chromista (three; 4%), Bryozoa (two; 3%), Ctenophora (two; 3%) and Porifera (one; 1%). Overall, the majority of horizon species are naturally present in Southeast Asia (29, 39%), followed by those present in the Americas (18, 24%), European coasts (10, 14%), central Asia (including the Black and Caspian seas; six, 8%), Africa (six, 8%), and with the remainder (five, 7%) from Australasia and the wider Indo‐Pacific or unknown (note that some species have a native range encompassing more than one of the above categories). Vectors (and associated pathways) of introduction were less certain than native origin from the literature available, but based on species characteristics (e.g*.* adhering species) and known introductions elsewhere, the following estimation of potential vectors for horizon species was noted: ballast water (39 potential incidences, 34%), followed by aquaculture (33, 28%), biofouling (31, 27%), aquarium trade (ten, 8%) and ‘other’ (three, 3%).

### 3.2 Outcomes and confidence

Following ROC curve analysis (Table 1) of the AS‐ISK scores (Table S3; Species Assessment Reports in S4), BRA thresholds could be computed successfully for all AS‐ISK taxonomic groups in the study (namely, brackish and marine fishes and lampreys, tunicates, marine invertebrates, marine Plantae and marine Protista), with the exception of brackish invertebrates due to low sample size (*n* = 4). Therefore, BRA and BRA+CCA thresholds were estimated for brackish and marine combined. Conversely, reliable calculations of individual BRA+CCA thresholds were not possible for marine Plantae and marine Protista due to their mean AUC values being <0.5, which was also the case for the combined marine Plantae and Protista threshold (see Section 2.4).

**Table 1:** Taxonomic aquatic organism group‐specific thresholds for the basic risk assessment (BRA) and BRA+CCA (climate change assessment) of the non‐native species (extant and horizon: see Tables S1 and S2, respectively) screened with AS‐ISK for the Inner and Middle RSA (see Table S3)

Aquatic organism group	BRA				BRA+CCA			
	Thr	AUC	LCI	UCI	Thr	AUC	LCI	UCI
Fishes and lampreys (brackish)	30.50	0.9592	0.8640	1.0000	22.50	0.8980	0.7103	1.0000
Fishes and lampreys (marine)	19.75	0.9286	0.8119	1.0000	21.75	0.7922	0.5475	1.0000
Tunicates	34.25	0.7656	0.4365	1.0000	34.25	0.9062	0.7018	1.0000
Invertebrates (brackish)^a^	26.25	0.7174	0.5753	0.8596	20.50	0.7207	0.5744	0.8671
Invertebrates (marine)	26.25	0.7348	0.5911	0.8786	20.50	0.7303	0.5766	0.8840
Plantae (marine)^b^	27.50	0.7857	0.5126	1.0000	28.25	0.6330	0.5344	0.7316
Protista (marine)^b^	28.50	0.6597	0.3824	0.9370	28.25	0.6330	0.5344	0.7316

Note

Mean, lower confidence interval (LCI) and upper confidence interval (UCI) for the Area Under the Curve (AUC) are provided.

Abbreviations: AS‐ISK, Aquatic Species Invasiveness Screening Kit; RSA, Regional Organization for Protection of Marine Environment Sea Area.

a BRA and BRA+CCA thresholds from combined brackish and marine invertebrates.

b BRA+CCA thresholds from all taxonomic groups combined.

All resulting AUCs (when using combinations of the thresholds described above) were above 0.5 (Table 1), indicating that AS‐ISK was able to discriminate reliably between non‐invasive and invasive species in the risk assessment area. Youden's *J* provided BRA thresholds ranging from 19.75 (marine fishes) to 34.25 (tunicates), and BRA+CCA thresholds from 20.5 (marine invertebrates and brackish invertebrates—the latter based on the combined groups) and 34.25 (tunicates). These group‐specific thresholds were therefore used for calibration of the risk outcomes at the species level, using the appropriate statistical use of interval brackets (‘]’ and ‘[‘; http://www.mathwords.com/i/interval_notation.htm). Accordingly, the BRA thresholds allowed the distinction of medium‐risk species with scores within the interval [1, Thr_BRA_[ from high‐risk species with scores within ]Thr_BRA_, 68]; and the BRA+CCA thresholds allowed distinction of medium‐risk species with scores within [1.0, Thr_BRA+CCA_[, from high‐risk species with scores within ]Thr_BRA+CCA_, 80]. Whereas species classified as low risk were those with BRA scores within [−20, 1[ and BRA+CCA scores within [−32, 1[.

**Figure 2:**
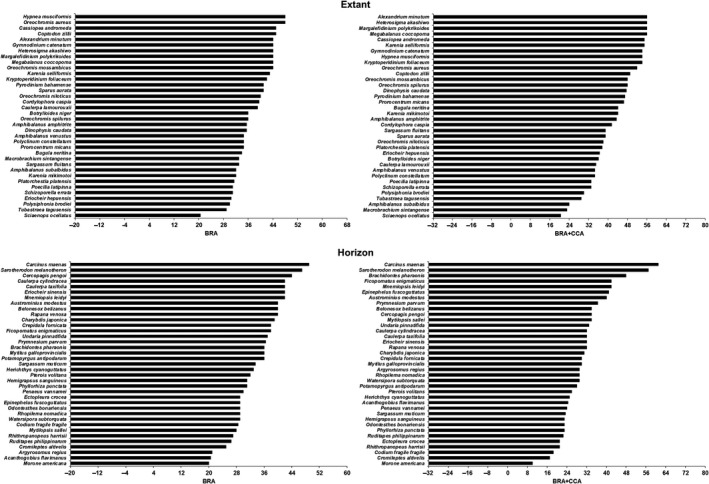
Ranking of extant (upper graphs) and horizon (lower graphs) non‐native species for the Regional Organization for Protection of the Marine Environment Sea Area that were attributed Aquatic Species Invasiveness Screening Kit scores at or above the threshold values (Table 1) for basic risk assessments (BRA) and BRA plus climate change assessments (BRA+CCA). For full details on all species and the assessment reports, see Tables [Link], [Link]

Of the 136 NNS assessed in total (i.e. extant and horizon: Table S3), based on the BRA thresholds, 73 (53.7%) were classified as high risk and 63 (46.3%) as medium risk (no low‐risk species); of the 85 species categorized a priori as invasive, 57 (67%) were classified as high risk (true positives) and 28 (33%) as medium risk; and of the 51 species categorized a priori as non‐invasive, 16 (31%) were classified as high risk (false positives) and 35 (69%) as medium risk. Based on the BRA+CCA thresholds, 81 (59.6%) species were classified as high risk, 50 (36.8%) as medium risk and five (3.7%) as low risk; of the 85 species categorized a priori as invasive, 61 (72%) have a high‐risk classification (true positives), 22 (26%) as medium risk and two (2%) as low risk (false positives: dark doto, *Doto kya* and nimble spray crab, *Percnon gibbesi*); and, of the 51 species categorized a priori as non‐invasive, 20 (39%) were classified as high‐risk species (false positives), 28 (55%) as medium risk and three (6%) as low risk (true negatives: charming aeolid *Microchlamylla amabilis*, mysid shrimp *Rhopalophthalmus tattersallae* and white‐crust cuthona *Trinchesia albocrusta*). The overview of AS‐ISK scores for species scoring at or above regional threshold for risk of invasiveness is given in Figure 2.

With regard to BRA scores, the highest‐scoring (invasive) NNS (score ≥45, taken as an ad hoc very high‐risk threshold value) were the green crab, *Carcinus maenas*, crozier weed *Hypnea musciformis*, blue tilapia *Oreochromis aureus*, blackchin tilapia *Sarotherodon melanotheron*, upside down jellyfish *Cassiopea andromeda* and redbelly tilapia *Coptodon zillii* (from higher to lower scores). As to BRA+CCA scores, the highest‐scoring (invasive) species (score ≥55, same criterion as per BRA) were *C. maenas*, *S. melanotheron*, *Alexandrium minutum*, *Heterosigma akashiwo*, *Margalefidinium polykrikoides*, titan acorn barnacle *Megabalanus coccopoma*, *C. andromeda* and *Karenia selliformis* (from higher to lower scores). There were no low‐risk NNS for the BRA, whereas for the BRA+CCA these included mysid shrimp, *R. tattersallae*, *D. kya*, *T. albocrusta*, *M. amabilis* and *P. gibbesi* (from lower to higher scores; Table S3).

The CCA increased the BRA score for 52 (38.2%) of the screened species, decreased it for 62 (45.6%) of them, and remained unchanged for the remaining 22 (16.2%; Table S3). Also, 15 (11.0%) of the screened species achieved the largest possible (positive) change in score of 12, and these included *C. maenas*, the highest‐scoring species for both the BRA and BRA+CCA (see above).

Mean CL (i.e. over all 55 Qs) was 2.71 ± 0.03 *SE*, mean CL_BRA_ 2.75 ± 0.03 *SE*, and mean CL_CCA_ 2.41 ± 0.06 *SE* (hence, in all cases indicating medium to high confidence). Also, there was a statistically significant Component × Status interaction (*F* ^#^_1,268_ = 22.85; *p* < .001; # = permutational value) and this was due to the mean CL_BRA_ being higher than mean CL_CCA_ (i.e*.* 2.78 vs. 2.07; *t* ^#^ = 7.81; *p* < .01) for the extant NNS, whereas for the horizon NNS, there were no significant differences detected (i.e. 2.73 vs. 2.65; *t* ^#^ = 0.84; *p* = .400). Mean CF (i.e. over all 55 Qs) was 0.678 ± 0.007 *SE*, mean CF_BRA_ = 0.687 ± 0.007 *SE*, and mean CF_CCA_ = 0.603 ± 0.016 *SE*. In all cases, the narrow standard errors indicated overall similarity in CLs and CFs across the NNS assessed.

Overall, the highest‐risk species had a BRA score of >45 or a combined BRA+CCA score of >55. For BRA, this included three (5%) of the extant NNS, and two (2.5%) of the horizon NNS. For combined BRA+CCA, this included five (9%) of the extant NNS and three (4%) of the horizon NNS.

## 4 DISCUSSION

Of the 56 extant NNS identified in the Inner and Middle RSA, 64% (36) of species were classified as likely to pose a risk of being invasive, and of the 80 horizon species, 46% (37) have attributes of risk of invasiveness. Of the species already present, the protozoan Chromista formed a majority at 25% (14 species) and had a high risk of invasiveness. In contrast to extant species, fish comprised the most common group of aquatic organisms of the horizon NNS forming 28% (22 species) of the total. Only three Chromista species were identified as horizon NNS, although further representatives of this taxonomic group may be found to be present in the Inner and Middle RSA in the future as they are poorly studied compared to other groups. In general, it is likely the current list of 136 species is only part of the non‐native biodiversity present in the risk assessment area and therefore should form a basis before further review by experts in‐region. Further study, particularly through field‐based monitoring, would likely reveal more NNS to be present.

For both extant and horizon NNS, ballast water was the most common introduction vector providing 51% of all instances of extant species introductions, and 34% for horizon species. This is not surprising given the high levels of shipping in the risk assessment area making this a prominent vector for NNS movement. Some 53,000 ships visit the Gulf annually in association with oil transportation alone (Al‐Yamani, Skryabin, & Durvasula, 2015), and in 2017 a total of 146,671 voyages were received into all ports within the Inner and Middle RSA (AIS data obtained on request from Marine Traffic: http://www.marinetraffic.com/en/p/ais-historical-data). This may also reflect the high percentage of Chromista identified as extant NNS, as ballast water is a common vector for the movement of these types of organism (Bailey, 2015; Gustaaf, 2015). The prominence of this vector and its link to transporting Chromista further highlights the potential for their low number identified in the horizon scanning to be an artefact of the formulation of the horizon list rather than the actuality. As one of the globally recognized and most important vectors for the introduction of NNS into aquatic systems, the Ballast Water Management Convention provides some legislative oversight to related activities (Olenin, Minchin, Daunys, & Zaiko, 2010). The ballast vector was followed by biofouling and aquaculture, with the latter being responsible for many of the fish species introductions.

The pathways associated with these introduction vectors for horizon species provide an indication of where management efforts should focus to reduce the likelihood of future introduction events. In addition, for those species likely to be transported by ship in ballast water or as hull foulants, the native range may provide an indication of likely ports of entry if matched to shipping pathways. However, this would only apply if the species has not already been introduced elsewhere and many species identified already have a wide Indo‐Pacific distribution. The initial vector and pathway analysis undertaken as part of risk screening for species could be strengthened by more in‐depth vector/pathway and hotspot analysis, focused particularly on shipping routes (international and regional—the latter important for the spread of NNS once introduced) and aquaculture.

The species identified as posing the highest risk of being invasive under current climatic conditions were the extant macro‐algal species, *H. musciformis* and the horizon crab species, *C. maenas*. These species are known to be transported *via* ballast water and to be invasive elsewhere. Also, *C. maenas* is found on several lists of global ‘top 100 invasive species’ (e.g. Lowe, Brown, Boudjelas, & De Poorter, 2000; O'Donnel, 2013), and consistent with this, these two species received the highest current (BRA) and future climate (BRA+CCA) scores of all species screened.

*Hypnea musciformis* is known to form dense floating algal mats (Russell, 1992), which can have socio‐economic impacts when they are washed ashore as they release noxious gases while decomposing (Russell, 1992). A study on the Hawaiian island of Maui estimated costs for ≈$20 million per year to manage the impacts of *H. musciformis* blooms, such as by cleaning rotting algae off beaches, reduction in property values and lost tourist revenues (Cesar, Van Beukering, Pintz, & Dierking, 2002). Ecologically, the species can outcompete other macro‐algae, and in Hawaii it has become the main food source of the green turtle *Chelonia mydas*. It is uncertain whether or not this alga is as nutritious as native species, and thus a dietary change could affect the fitness of the turtle population (Russell & Balazs, 1994), adding to other pressures affecting turtle populations in the RSA (Pilcher et al., 2014).

*Carcinus maenas* is a generalist known to exert adverse impacts on marine ecosystems, including socially and economically important native species such as crabs and shellfish. A major example includes the collapse of the New England shellfish industry in the 1950s resulting from the introduction of *C. maenas* (Smith, Baptist, & Chin, 1955). Its impacts on aquaculture productivity over the west coast of the USA caused severe economic loses at an estimated $44 million (Klassen & Locke, 2007). In addition, this shore crab can cause adverse ecological impacts due to habitat degradation, including alterations to the structure of intertidal and subtidal communities (Cohen, Carlton, & Fountain, 1955). For example, extensive foraging behaviour of the crab has shown to be a major cause of the significant declines in eelgrass *Zostera marina* beds in the Gulf of St. Lawrence, Nova Scotia (Garbary, Miller, Williams, & Seymour, 2014). In another example, *C. maenas* was responsible for the decline of the native Dungeness crab *Metacarcinus magister* on the west coast USA through monopolizing prey resources owing to their greater claw strength (Yamada, Davidson, & Fisher, 2010). The impacts identified elsewhere for these high‐risk species highlight the need for effective management of NNS in the Inner and Middle RSA from environmental, social and economic perspectives.

Climate changes predicted for the RSA, while potentially reducing the risk of establishment of many NNS, may benefit coliform bacteria and dinoflagellates (Van Lavieren et al., 2011). The BRA+CCA score increased compared to the initial BRA score for 52 species of all 136 screened, suggesting some species may have a greater risk of invasion with predicted climate change. However, the risk of being invasive was reduced for 62 species, as the naturally extreme conditions of the region are already at the upper end of species' temperature tolerance and increasing temperatures would only exacerbate this stress. In accordance with climate predictions for the RSA (Van Lavieren et al., 2011), the screenings undertaken in this study suggest that the majority of Chromista are likely to pose an increased risk under future conditions (i.e. 71% of all Chromista across extant and horizon species), with increased risk also anticipated for some other taxon groups, namely fish (47% increase risk of invasion in response to climate change), invertebrates (25%) and, to a minor degree, plants (4%). For the other aquatic organism groups, a majority of species would decline in risk as a result of climate change. These contrasting predictions highlight the likelihood of unforeseen responses by species to climate change, and detailed climate modelling for the risk assessment area would enable a more detailed understanding of risks posed by NNS (in particular those species whose BRA+CCA score increased) as well as identifying locations of potentially higher risk based on climate variables. Also, invasiveness risk response to climate change may vary between the Inner and Middle RSA, as these have different climate parameters due to their oceanography (Riefl et al., 2012; Van Lavieren et al., 2011; Vaughan et al., 2019).

The present study represents the first application of AS‐ISK in the Inner and Middle RSA and the first application of this decision‐support tool anywhere to a multi‐taxonomic study looking at extant and horizon species. The medium‐to‐high CLs of the screenings and the ability to provide regional thresholds for some taxonomic groups are of particular note, as this highlights the increased specificity to the results, which is important in a region where species are considered generally less likely to establish due to the locally extreme climatic conditions. Overall, the present results suggest that AS‐ISK is a useful and valid decision‐support tool for identifying potentially invasive species, both extant and horizon, and assist decision‐makers in setting priorities for NNS management. The present study complements other AS‐ISK based assessments of NNS undertaken in wider Arabia and the eastern Mediterranean (i.e. Bilge, Filiz, Yapici, Tarkan, & Vilizzi, 2019; Tarkan, Sarı, et al., 2017; Tarkan, Vilizzi, et al., 2017), further highlighting the usefulness of AS‐ISK for NNS management in the Inner and Middle RSA. It also provides wider validation of the ability of AS‐ISK to identify NNS risk in a variety of aquatic environments, including those with more specialized and extreme climatic conditions, as well as to assist in NNS management of both extant and horizon species.

The present AS‐ISK assessments also helped identify gaps in knowledge with regard to the types and magnitude of adverse impacts that could be imposed on the Inner and Middle RSA. Understanding the impacts already caused in the latter or elsewhere by specific NNS can help to identify where similar impacts may occur in the future, and thus enable preventative steps to be taken to reduce these in advance. An example is the use of early warning systems to monitor algal blooms caused by Chromista to enable the movement or closure of aquaculture farms, or the harvest of their outputs in advance to reduce risk to human health (see FAO, 2017). Such warning systems could be particularly relevant in the Inner and Middle RSA, as in September–October 1999 a harmful algal bloom primarily composed of *K. selliformis* (a cryptogenic NNS) and *Prorocentrum rhathymum* (a definite NNS) caused significant mortality of wild and farmed fish in Kuwait Bay resulting in an estimated economic loss of $7 million (Al‐Yamani, Saburova, & Polikarpov, 2012). Another NNS Chromista *Gymnodinium catenatum* in the Inner and Middle RSA is known elsewhere around the world to have caused paralytic shellfish poisoning, which can have significant human health implications (Hoagland, Anderson, & White, 2002).

The present study has also highlighted key species and taxonomic groups with high risk of being/becoming invasive that should provide a focus for further regional study and monitoring. Linked to impact management, two of the key taxonomic groups identified were Chromista and fish (many of which are transported via aquaculture). Further study could include in‐region surveys to detect the presence and establish the current distribution of extant species, and to monitor for horizon species. Such work could use well‐established taxonomic survey methods, environmental DNA methods and regular monitoring of vectors (Trebitz et al., 2017), for example, vessel hulls and ballast water. This will help identify the exact NNS present (i.e. provide ground‐truthing of species lists) and their current distribution.

In addition to monitoring, NNS vector (and associated pathway) management should be put into place, including ensuring compliance with the Ballast Water Management Convention for vessels entering the Inner and Middle RSA and in wider port management practices; and implementation of IMO guidelines for the control and management of ships' biofouling to minimize the transfer of invasive aquatic species (Biofouling Guidelines and resolution MEPC.207(62), 2011). This would include ensuring relevant vessels entering the Inner and Middle RSA have ballast water management plans in place, adequate treatment of ballast water to reduce biological organism survival occurring within vessel systems (e.g. use of ozone, UV and other forms of filtration), and ballast water exchange occurring in deep waters (away from coastal waters; IMO, 2018). Furthermore, in‐water cleaning of vessel hulls should be minimized where possible or scrapings adequately captured and disposed of on‐land (Hopkins & Forrest, 2008).

Non‐native species management in aquaculture should be established within existing and future biosecurity measures including ensuring stock does not come from areas with NNS present that are known to be transported *via* aquaculture (e.g. as hitchhikers). Reducing the use of NNS in aquaculture unless they are going to be farmed in enclosed facilities should also be an aim of the management and wider policy regarding the aquaculture vector and its associated pathways. A good overview of existing global regulations, guidelines and methods for reducing the risk and impact of NNS in aquaculture is provided by Hewitt, Campbell, and Gollasch (2006).

Surveys and monitoring combined with further vector/pathway analysis and climate modelling, as suggested within wider discussion above, would enable identification of hotspots of invasion more generally, allowing a geographical as well as species‐specific focus to monitoring and management efforts and help to understand better the spread potential within the Inner and Middle RSA for extant NNS. Targeted management of vectors and pathways will help reduce the risk of NNS being introduced in the first instance and combined with monitoring of species themselves, enabling rapid response processes to newly identified introduction events to reduce risk of establishment and spread. This is in line with the CBD guiding principles of prevention of NNS introductions being preferable, followed by early identification and rapid response to reduce establishment and eradication as a last resort. Ensuring NNS are identified and managed before they become established and potentially invasive is especially important in regions where the sensitivity of existing environments to increased pressures is high. Overall, effective NNS management will help provide another step towards protecting the unique marine and brackish water environments of the RSA alongside existing environmental management measures.

In conclusion, the present study provides baseline knowledge of NNS present in the Inner and Middle RSA and, for the first time, identifies those with the potential to become invasive in the future. This is important as the RSA experiences unique and extreme climatic conditions that are predicted to become harsher to aquatic organisms with climate change. As many species are already at their limits of tolerance, the impacts of invasive NNS combined with existing pressures could increase the risk of species and habitat loss and degradation in the region. Therefore, it is vital to understand the baseline risk that NNS pose to the RSA both now and in the future.

## CONFLICT OF INTEREST

The authors of this manuscript have no conflicts of interest to declare.

## Data Availability

The data that support the findings of this study are available in the Supporting Information of this article.
