# Supplementary material for: Identifying potentially invasive non‐native marine and brackish water species for the Arabian Gulf and Sea of Oman
Source: Glob Chang Biol. 2020 Feb 4;26(4):2081–92. doi: 10.1111/gcb.14964 (PMC7154788; doi:10.1111/gcb.14964)
Supplement: Supplementary file 1 [file GCB-26-2081-s001.docx]

# Supplementary material

Includes Tables S1, S2, S3 and reference list for Table S1

**Table S1** Extant non-native species (with corresponding Order and Family) screened for their risk of invasiveness in the Inner and Middle Regional Organization for the Protection of the Marine Environment (ROPME) Sea Area (RSA) with the Aquatic Species Invasiveness Screening Kit (AS-ISK) and arranged by AS-ISK taxonomic groups. Countries where records for the species are known and associated references are provided, along with potential pathways of introduction. Full references in Supplementary Information.

| **Taxon name** | **Common name** | **Countries** | **References** | **Pathways** |
| --- | --- | --- | --- | --- |
| **Fishes and lampreys (brackish)** | | | | |
| *Coptodon zillii* | redbelly tilapia | Iran, Iraq, Saudi Arabia | Al-Faisal *et al*. (2014); Khaefi *et al*. (2014) | Aquaculture; Aquarium trade; Mosquito (Biological) control |
| *Oreochromis aureus* | blue tilapia | Iraq, Kuwait, Oman, Saudi Arabia, UAE | Al-Faisal *et al*. (2014); Al-Faisal & Mutlak (2014); Bartley (2006); Canonico *et al*. (2005); Lin & Suresh (1992); McDonald (1987); Siddiqui & Al-Harbi (1995); Teimori *et al*. (2017); Valkhania *et al*. (2016); Victor & Makki (2000) | Aquaculture |
| *Oreochromis mossambicus* | Mozambique tilapia | Kuwait, Oman, Qatar, Saudi Arabia, UAE | Froese & Pauly (2004); Norman *et al*. (2009); Pullin *et al*. (1997) | Aquaculture |
| *Oreochromis niloticus* | Nile tilapia | Iran, Kuwait, Qatar, Saudi Arabia, UAE | Al-Faisal & Mutlak (2014); Bartley (2006); Coad (1995); Froese & Pauly (2004); Pullin *et al*. (1997); Siddiqui *et al*. (1989) | Aquaculture |
| *Oreochromis spilurus* | Sabaki tilapia | Kuwait, Saudi Arabia, UAE | Cruz *et al*. (1990); El-Sayed (2006); Jonassen *et al*. (1997); Sherly & Sambhu (2016) | Aquaculture |
| *Poecilia latipinna* | sailfin molly | Iraq, Oman, Saudi Arabia | Alkahem *et al*. (2007); Al-Faisal *et al*. (2014); Bartley (2006); Koutsikos *et al*. (2018) | Aquarium trade |
| *Rhinogobius brunneus* | Amur goby | Iran, Kuwait | Al-Hassan & Miller (1987); Al-Yamani *et al*. (2015) | Ballast water; Aquarium trade |
| *Sarotherodon galilaeus* | mango tilapia | Iraq, Oman | Bartley (2006) | Aquaculture; Mosquito (Biological) control |
| **Fishes and lampreys (marine)** | | | | |
| *Sciaenops ocellatus* | red drum | UAE | Gonzalez-Felix *et al*. (2018) | Aquaculture |
| *Sparus aurata* | gilthead seabream | Bahrain, Kuwait, Oman, Saudi Arabia, UAE | Al-Yamani *et al*. (2015) | Aquaculture |
| **Tunicates** | | | | |
| *Botrylloides niger* | – | Bahrain | Naser (2017) | Biofouling - other |
| *Ciona intestinalis* | sea vase | Oman | Dobretsov (2015) | Ballast water |
| *Diplosoma listerianum* | gray encrusting compound tunicate | Bahrain | Naser (2017) | Biofouling - other |
| *Microcosmus squamiger* | scaly tunicate | Oman | Meliane *et al*. (2001) | Ballast water; Hull fouling |
| *Polyclinum constellatum* | – | Bahrain | Naser (2017) | Biofouling - other |
| *Styela plicata* | pleated sea squirt | Oman, Saudi Arabia | Khaleghi (2016); Saad (2016) | Hull fouling |
| *Symplegma brakenhielmi* | – | Bahrain | Naser (2017) | Biofouling - other |
| **Invertebrates (brackish)** | | | | |
| *Amphibalanus subalbidus* | – | Iran | Naser *et al*. (2015); Shahdadi *et al*. (2014) | Biofouling - other |
| *Cordylophora caspia* | freshwater hydroid | Kuwait, Iraq | Arndt (1989); Pagad *et al*. (2018) | Ballast water |
| **Invertebrates (marine)** | | | | |
| *Amphibalanus amphitrite* | purple acorn barnacle | Bahrain, Kuwait, Iran | Naser (2017); Naser *et al*. (2015); Shahdadi *et al*. (2014); | Ballast water; Hull fouling |
| *Amphibalanus improvisus* | – | Iran, Kuwait | Naser *et al*. (2015); Nasrolahi (2007); Nasrolahi *et al*. (2006); Shahdadi *et al*. (2014) | Ballast water; Hull fouling |
| *Amphibalanus venustus* | – | Bahrain, Kuwait, Iran | Naser *et al*. (2015); Shahdadi *et al.* (2014) | Ballast water; Hull fouling |
| *Bugula neritina* | branching moss worm | Oman | Dobretsov (2015); Jones (1986); Naser (2017); Pagad *et al*. (2018) | Biofouling - other |
| *Bugulina stolonifera* | – | Oman | Jones (1986); Pagad *et al*. (2018) | Biofouling - other |
| *Cassiopea andromeda* | upside-down jellyfish | Iran | Mohebbi *et al*. (2018); Nabipour *et al*. (2015) | Ballast water |
| *Doto kya* | dark doto | Kuwait | Al-Yamani *et al*. (2014, 2015) | Ballast water |
| *Eriocheir hepuensis* | Hepu mitten crab | Iran, Iraq, Kuwait, Qatar | Clark *et al*. (2006); Hashim, (2010); Naderloo, (2014); Naser *et al*. (2012) | Ballast water |
| *Leostyletus misakiensis* | Misaki balloon aeolis | Kuwait | Al-Yamani *et al*. (2014, 2015) | Ballast water |
| *Macrobrachium sintangense* | rockpool prawn | Iran, Iraq, Kuwait | Dore & Frimodt, (1987); Fotonov *et al*. (2014); Holthuis & Hassan, (1975) | Ballast water |
| *Megabalanus coccopoma* | titan acorn barnacle | Iran | Shahdadi *et al.* (2014) | Biofouling - other |
| *Microchlamylla amabilis* | charming aeolid | Kuwait | Al-Yamani *et al*. (2014, 2015) | Ballast water |
| *Platorchestia platensis* | – | Bahrain | Naser (2017) | Ballast water |
| *Pseudodiaptomus ardjuna* | – | Iraq, Kuwait | Mohamed, (2011) | Ballast water |
| *Rhopalophthalmus tattersallae* | mysid shrimp | Kuwait | Al-Yamani *et al*. (2015); Bollens *et al*. (2002); Carlton & Geller (1993); Grabe (1989); WHO (1997) | Ballast water |
| *Schizoporella errata* | branching bryozoan | Kuwait | Uddin *et al*. (2017) | Hull fouling |
| *Trinchesia albocrusta* | white-crust cuthona | Kuwait | Al-Yamani *et al*. (2015) | Ballast water |
| *Tubastraea tagusensis* | ahermatypic corals | Kuwait | Creed *et al*. (2017) | Biofouling - other |
| **Plantae (marine)** | | | | |
| *Caulerpa lamourouxii* | coarse seagrape | UAE | Klein & Verlaque (2007); Vanneyre *et al*. (2014) | Ballast water; Aquarium trade |
| *Grateloupia filicina* | Agardh chop-chop | Kuwait | Al-Yamani *et al*. (2014, 2015) | Ballast water |
| *Hypnea musciformis* | crozier weed | Oman | Pagad *et al*. (2018); Silva *et al*. (1996); Sohrabipour & Rabii (1999) | Ballast water |
| *Polysiphonia brodiei* | – | Iran, Kuwait, Qatar, Saudi Arabia | Al-Yamani *et al*. (2014); John & Al-Thani (2014); Pagad *et al*. (2018); Silva *et al*. (1996) | Ballast water; Hull fouling |
| *Ulva ohnoi* | – | Bahrain, Iran, Iraq, Kuwait, Oman, Qatar, Saudi Arabia, UAE | Pirion *et al*. (2016) | Ballast water; Biofouling - other |
| **Protista (marine)** | | | | |
| *Alexandrium minutum* | – | Kuwait | Gilbert *et al*. (2002) | Ballast water |
| *Dinophysis caudata* | – | UAE | Subrahmanyam & Samar (1961: in Singh *et al*. 2014) | Ballast water |
| *Gymnodinium catenatum* | – | Kuwait, UAE | Glibert *et al*. (2002); Hallegraeff & Bolch (1992); Hallegraeff *et al*. (1995); Heil *et al*. (2001); Pagad *et al*. (2018) | Ballast water |
| *Heterosigma akashiwo* | – | Iran, Kuwait, Saudi Arabia | Al-Yamani *et al*. (2014, 2015); Engesmo *et al*. (2016); Shapoori & Gholami, (2014) | Ballast water |
| *Karenia mikimotoi* | – | UAE | IUCN (2016) | Ballast water |
| *Karenia selliformis* | – | Kuwait | Al-Yamani *et al*. (2015); Heil *et al*. (2001) | Ballast water |
| *Kryptoperidinium foliaceum* | – | Kuwait | Saburova *et al*. (2012) | Ballast water |
| *Margalefidinium polykrikoides* | – | Iran, Kuwait, Oman, UAE | Aein-Jamshid *et al*. (2014); Al-Azri *et al*. (2015); Al-Yamani *et al*. (2014); Hallegraeff, (2015); Richlen *et al*. (2010); Rountos *et al.* (2017) | Ballast water |
| *Myrionema orbiculare* | – | Kuwait | Al-Yamani *et al*. (2014, 2015) | Aquaculture; Ballast water |
| *Prorocentrum mexicanum* | – | Iran, Kuwait | Al-Yamani *et al*. (2012) | Ballast water |
| *Prorocentrum micans* | – | Kuwait, UAE | Ibrahim & Al-Shawi (2015) | Ballast water |
| *Pyrodinium bahamense* | – | Kuwait, UAE | Usup *et al*. (2012); Bohm (1931) | Ballast water |
| *Sargassum muticum* | jap weed | Iran, Kuwait | Sohrabipour & Rabii (1999) | Aquaculture; Ballast water; Biofouling - other |
| *Tintinnopsis ampla* | – | Kuwait | Al-Yamani *et al*. (2015); Saburova *et al*. (2012) | Ballast water |

**Table S2** Horizon non-native species (with corresponding Order and Family) screened for their risk of invasiveness in the in the Inner and Middle RSA with AS-ISK and arranged by AS-ISK taxonomic groups. Potential pathways of introduction are indicated.

| **Group/Taxon name** | | **Common name** | **Pathways** |
| --- | --- | --- | --- |
| **Fishes and lampreys (brackish)** | | | |
| *Belonesox belizanus* | | Pike killifish | Aquarium trade |
| *Clupeonella cultriventris* | | Black and Caspian Sea sprat | Aquaculture; Ballast water |
| *Herichthys cyanoguttatus* | | Rio Grande cichlid | Aquarium trade |
| *Mayaheros uropthalmus* | | Mexican moharra | Aquarium trade |
| *Proterorhinus marmoratus* | | eastern tubenose goby | Ballast water; Biofouling - other |
| *Tilapia mariae* | | spotted tilapia | Aquaculture |
| **Fishes and lampreys (marine)** | | | |
| *Acanthogobius flavimanus* | | yellowfin goby | Ballast Water; Hull fouling |
| *Acanthopagrus latus* | | yellowfin seabream | Aquaculture |
| *Acentrogobius pflaumii* | | striped sandgoby | Ballast water |
| *Argyrosomus regius* | | meagre | Aquaculture |
| *Ascidiella aspersa* | | dirty sea squirt | Aquaculture; Aquarium trade; Ballast water; Hull fouling |
| *Botrylloides perspicuus* | | – | Aquaculture; Hull fouling |
| *Botrylloides violaceus* | | purple colonial tunicate | Aquaculture; Ballast water; Hull fouling |
| *Chelon auratus* | | golden grey mullet | Aquaculture |
| *Chelon ramada* | | thinlip grey mullet | Aquaculture |
| *Cromileptes altivelis* | | humpback grouper | Intentional release - other |
| *Dicentrarchus labrax* | | European seabass | Aquaculture |
| *Epinephelus fuscoguttatus* | | brown-marbled grouper | Aquaculture; Aquarium trade |
| *Lucania parva* | | rainwater killifish | Aquaculture |
| *Morone americana* | | white perch | Ballast water |
| *Odontesthes bonariensis* | | Argentinian silverside | Aquaculture |
| *Peristedion cataphractum* | | African armoured searobin | Aquaculture |
| *Polyandrocarpa zorritensis* | | – | Aquaculture; Ballast water; Hull fouling |
| *Pterois volitans* | | red lionfish | Aquarium trade; Ballast water |
| *Sarotherodon melanotheron* | | blackchin tilapia | Aquaculture |
| *Styela clava* | | clubbed tunicate | Aquaculture; Hull fouling |
| *Taractichthys longipinnis* | | big-scale pomfret | Aquaculture |
| **Invertebrates (brackish)** | | | |
| *Ficopomatus enigmaticus* | | Australian tubeworm | Ballast water; Hull fouling |
| *Limnoperna fortunei* | | Chinese freshwater mussel | Ballast water; Hull fouling |
| **Invertebrates (marine)** |  |  |  |
| *Acartia (Acanthacartia) tonsa* | | ‘Hankajalkaisäyriäinen’ | Ballast water |
| *Alitta succinea* | | – | Hull fouling |
| *Arcuatula senhousia* | | green mussel | Hull fouling |
| *Austrominius modestus* | | Australian barnacle | Ballast water; Hull fouling |
| *Beroe ovata* | | brown comb jelly | Biological control |
| *Brachidontes pharaonis* | | – | Ballast water; Hull fouling |
| *Carcinus maenas* | | green crab | Ballast water |
| *Carijoa riisei* | | snowflake coral | Hull fouling |
| *Cercopagis pengoi* | | fish-hook waterflea | Ballast water; Hull fouling |
| *Charybdis (Charybdis) japonica* | | Japanese swimming crab | Ballast water |
| *Crassostrea virginica* | | American cupped oyster | Aquaculture |
| *Crepidula fornicata* | | slipper limpet | Aquaculture |
| *Ectopleura crocea* | | pink-mouth hydroid | Hull fouling |
| *Eriocheir sinensis* | | Chinese mitten crab | Ballast water |
| *Eualetes tulipa* | | – | Hull fouling |
| *Gonionemus vertens* | | clinging jellyfish | Ballast Water |
| *Hediste diversicolor* | | ragworm | Aquaculture; Intentional Release - other |
| *Hemigrapsus sanguineus* | | Asian shore crab | Ballast water; Hull fouling |
| *Hemigrapsus takanoi* | | Brush clawed shore crab | Ballast water; Hull fouling |
| *Marenzelleria neglecta* | | bristleworm | Ballast water; Hull fouling |
| *Mnemiopsis leidyi* | | warty comb jelly | Ballast water; Hull fouling |
| *Mycale (Mycale) grandis* | | orange keyhole sponge | Hull fouling |
| *Mytilopsis sallei* | | Santo Domingo falsemussel | Ballast water; Hull fouling |
| *Mytilus galloprovincialis* | | Mediterranean mussel | Aquaculture; Ballast water; Hull fouling |
| *Palaemon macrodactylus* | | oriental shrimp | Aquaculture |
| *Penaeus japonicus* | | kuruma prawn | Aquaculture |
| *Penaeus vannamei* | | whiteleg shrimp | Aquaculture |
| *Percnon gibbesi* | | nimble spray crab | Hull fouling |
| *Perna perna* | | South American rock mussel | Ballast water |
| *Petricolaria pholadiformis* | | false angel wing | Aquaculture |
| *Phyllorhiza punctata* | | Australian spotted jellyfish | Ballast water; Hull fouling |
| *Pileolaria berkeleyana* | | – | Hull fouling |
| *Potamocorbula amurensis* | | brackish-water corbula | Ballast Water |
| *Potamopyrgus antipodarum* | | New Zealand mudsnail | Aquarium trade; Ballast water |
| *Pseudodiaptomus marinus* | | – | Aquaculture |
| *Rapana venosa* | | purple whelk | Ballast water |
| *Rhithropanopeus harrisii* | | Harris mud crab | Ballast water |
| *Rhopilema nomadica* | | – | Ballast water |
| *Ruditapes philippinarum* | | Manila clam | Aquaculture |
| *Tricellaria inopinata* | | – | Aquaculture; Hull fouling |
| *Watersipora subtorquata* | | – | Aquaculture; Hull fouling |
| **Plantae (marine)** | | | |
| *Caulerpa cylindracea* | | – | Aquarium trade |
| *Caulerpa taxifolia* | | green sea palm | Aquarium trade |
| *Codium fragile fragile* | | sponge seaweed | Ballast water |
| *Eucheuma spp.* | | – | Aquaculture |
| *Gracilaria tikvahiae* | | – | Aquaculture; Aquarium trade |
| *Grateloupia turuturu* | | – | Aquaculture; Ballast water; Hull fouling |
| *Ulva australis* | | – | Ballast water; Biofouling - other |
| **Protista (marine)** | | | |
| *Prymnesium parvum* | | – | Ballast water |
| *Sargassum fluitans* | | gulf weed | Aquaculture; Ballast water; Hull fouling |
| *Undaria pinnatifida* | | wakame | Ballast water |

**Table S3** Non-native species assessed with AS-ISK for the Inner and Middle RSA. For each species, the status (E = extant; H = horizon: see Tables S1 and S2, respectively), *a priori* categorisation for invasiveness (N = non-invasive; Y = invasive), BRA and BRA+CCA scores and corresponding Risk outcomes based on aquatic organism group-specific (or combined) thresholds (Thr: see Table 1), difference (Delta) between BRA+CCA and BRA scores, Confidence Level (CL), Confidence Factor (CF) (see main text for explanation) for all questions (Total) and separately for the BRA and CCA components of the risk assessment are provided. Risk outcomes as follows: Low (L) = score within [−20, 1[ for the BRA and within [−32, 1[ for the BRA+CCA; Medium (M) = score within [1, Thr[; High (H) = score within ]Thr, 68] for the BRA and within ]Thr, 80] for the BRA+CCA. Note that thresholds reported hereafter are presented using the appropriate statistical use of interval brackets: ‘]’ and ‘[’ ([www.mathwords.com/i/interval_notation.htm](http://www.mathwords.com/i/interval_notation.htm)). Aquatic organism group IDs after AS-ISK coding: 6 = Fishes and lampreys (brackish); 7 = Fishes and lampreys (marine); 8 = Tunicates; 11 = Invertebrates (brackish); 12 = Invertebrates (marine); 18 = Plantae (marine); 21 = Protista (marine).

|  |  |  |  | **BRA** | | |  | **BRA+CCA** | | |  | **CL** | | |  | **CF** | | |
| --- | --- | --- | --- | --- | --- | --- | --- | --- | --- | --- | --- | --- | --- | --- | --- | --- | --- | --- |
| **ID** | **Taxon name** | **Status** | ***A priori*** | **Score** | **Thr** | **Risk** |  | **Score** | **Thr** | **Risk** | **Delta** | **Total** | **BRA** | **CCA** |  | **Total** | **BRA** | **CCA** |
| 6 | *Belonesox belizanus* | H | Y | 40.0 | 30.50 | H |  | 34.0 | 22.50 | H | −6.0 | 3.1 | 3.1 | 2.8 |  | 0.78 | 0.79 | 0.71 |
| 6 | *Clupeonella cultriventris* | H | N | 18.0 | 30.50 | M |  | 12.0 | 22.50 | M | −6.0 | 2.7 | 2.7 | 2.7 |  | 0.66 | 0.66 | 0.67 |
| 6 | *Coptodon zillii* | E | Y | 45.0 | 30.50 | H |  | 49.0 | 22.50 | H | 4.0 | 3.1 | 3.1 | 2.5 |  | 0.77 | 0.79 | 0.63 |
| 6 | *Herichthys cyanoguttatus* | H | Y | 33.0 | 30.50 | H |  | 25.0 | 22.50 | H | −8.0 | 3.0 | 2.9 | 4.0 |  | 0.75 | 0.72 | 1.00 |
| 6 | *Mayaheros uropthalmus* | H | N | 30.0 | 30.50 | M |  | 20.0 | 22.50 | M | −10.0 | 2.9 | 2.8 | 3.8 |  | 0.73 | 0.70 | 0.96 |
| 6 | *Oreochromis aureus* | E | Y | 48.0 | 30.50 | H |  | 52.0 | 22.50 | H | 4.0 | 3.3 | 3.4 | 2.3 |  | 0.83 | 0.86 | 0.58 |
| 6 | *Oreochromis mossambicus* | E | Y | 44.0 | 30.50 | H |  | 48.0 | 22.50 | H | 4.0 | 3.3 | 3.4 | 2.2 |  | 0.82 | 0.86 | 0.54 |
| 6 | *Oreochromis niloticus* | E | Y | 40.0 | 30.50 | H |  | 38.0 | 22.50 | H | −2.0 | 3.1 | 3.2 | 2.3 |  | 0.78 | 0.80 | 0.58 |
| 6 | *Oreochromis spilurus* | E | N | 36.0 | 30.50 | H |  | 48.0 | 22.50 | H | 12.0 | 3.1 | 3.1 | 3.0 |  | 0.77 | 0.77 | 0.75 |
| 6 | *Poecilia latipinna* | E | Y | 31.0 | 30.50 | H |  | 33.0 | 22.50 | H | 2.0 | 2.2 | 2.3 | 1.2 |  | 0.54 | 0.57 | 0.29 |
| 6 | *Proterorhinus marmoratus* | H | N | 29.5 | 30.50 | M |  | 17.5 | 22.50 | M | −12.0 | 2.4 | 2.3 | 3.2 |  | 0.59 | 0.57 | 0.79 |
| 6 | *Rhinogobius brunneus* | E | N | 12.5 | 30.50 | M |  | 12.5 | 22.50 | M | 0.0 | 1.7 | 1.8 | 1.0 |  | 0.43 | 0.45 | 0.25 |
| 6 | *Sarotherodon galilaeus* | E | N | 29.0 | 30.50 | M |  | 25.0 | 22.50 | H | −4.0 | 2.9 | 3.0 | 2.0 |  | 0.73 | 0.76 | 0.50 |
| 6 | *Tilapia mariae* | H | N | 18.0 | 30.50 | M |  | 6.0 | 22.50 | M | −12.0 | 3.1 | 3.0 | 4.0 |  | 0.78 | 0.76 | 1.00 |
| 7 | *Acanthogobius flavimanus* | H | Y | 20.5 | 19.75 | H |  | 24.5 | 21.75 | H | 4.0 | 2.9 | 2.9 | 2.5 |  | 0.72 | 0.73 | 0.63 |
| 7 | *Acanthopagrus latus* | H | N | 8.0 | 19.75 | M |  | 12.0 | 21.75 | M | 4.0 | 2.3 | 2.3 | 2.0 |  | 0.57 | 0.58 | 0.50 |
| 7 | *Acentrogobius pflaumii* | H | N | 19.5 | 19.75 | M |  | 25.5 | 21.75 | H | 6.0 | 2.5 | 2.6 | 1.7 |  | 0.62 | 0.65 | 0.42 |
| 7 | *Argyrosomus regius* | H | N | 21.0 | 19.75 | H |  | 29.0 | 21.75 | H | 8.0 | 2.5 | 2.5 | 2.3 |  | 0.62 | 0.63 | 0.58 |
| 7 | *Chelon auratus* | H | N | 12.0 | 19.75 | M |  | 16.0 | 21.75 | M | 4.0 | 2.6 | 2.7 | 1.8 |  | 0.65 | 0.68 | 0.46 |
| 7 | *Chelon ramada* | H | N | 19.0 | 19.75 | M |  | 25.0 | 21.75 | H | 6.0 | 2.5 | 2.5 | 2.7 |  | 0.63 | 0.63 | 0.67 |
| 7 | *Cromileptes altivelis* | H | N | 25.0 | 19.75 | H |  | 17.0 | 21.75 | M | −8.0 | 2.6 | 2.6 | 2.8 |  | 0.65 | 0.64 | 0.71 |
| 7 | *Dicentrarchus labrax* | H | N | 13.0 | 19.75 | M |  | 17.0 | 21.75 | M | 4.0 | 2.7 | 2.8 | 2.2 |  | 0.68 | 0.70 | 0.54 |
| 7 | *Epinephelus fuscoguttatus* | H | Y | 29.0 | 19.75 | H |  | 41.0 | 21.75 | H | 12.0 | 2.7 | 2.8 | 2.7 |  | 0.69 | 0.69 | 0.67 |
| 7 | *Lucania parva* | H | N | 11.0 | 19.75 | M |  | 17.0 | 21.75 | M | 6.0 | 2.6 | 2.6 | 2.8 |  | 0.65 | 0.64 | 0.71 |
| 7 | *Morone americana* | H | Y | 20.0 | 19.75 | H |  | 10.0 | 21.75 | M | −10.0 | 2.9 | 2.8 | 3.8 |  | 0.73 | 0.70 | 0.96 |
| 7 | *Odontesthes bonariensis* | H | Y | 29.0 | 19.75 | H |  | 23.0 | 21.75 | H | −6.0 | 2.7 | 2.7 | 2.7 |  | 0.67 | 0.67 | 0.67 |
| 7 | *Peristedion cataphractum* | H | N | 2.0 | 19.75 | M |  | 6.0 | 21.75 | M | 4.0 | 2.4 | 2.5 | 2.0 |  | 0.61 | 0.62 | 0.50 |
| 7 | *Pterois volitans* | H | Y | 32.0 | 19.75 | H |  | 26.0 | 21.75 | H | −6.0 | 2.8 | 2.8 | 2.8 |  | 0.69 | 0.69 | 0.71 |
| 7 | *Sarotherodon melanotheron* | H | Y | 47.0 | 19.75 | H |  | 57.0 | 21.75 | H | 10.0 | 3.2 | 3.2 | 3.2 |  | 0.80 | 0.80 | 0.79 |
| 7 | *Sciaenops ocellatus* | E | N | 20.5 | 19.75 | H |  | 20.5 | 21.75 | M | 0.0 | 2.1 | 2.2 | 1.0 |  | 0.53 | 0.56 | 0.25 |
| 7 | *Sparus aurata* | E | Y | 41.0 | 19.75 | H |  | 39.0 | 21.75 | H | −2.0 | 2.0 | 2.2 | 1.0 |  | 0.51 | 0.54 | 0.25 |
| 7 | *Taractichthys longipinnis* | H | N | 6.0 | 19.75 | M |  | 6.0 | 21.75 | M | 0.0 | 2.3 | 2.4 | 1.0 |  | 0.57 | 0.61 | 0.25 |
| 8 | *Ascidiella aspersa* | H | Y | 23.5 | 34.25 | M |  | 15.5 | 34.25 | M | −8.0 | 3.1 | 3.2 | 3.0 |  | 0.79 | 0.79 | 0.75 |
| 8 | *Botrylloides niger* | E | N | 36.0 | 34.25 | H |  | 36.0 | 34.25 | H | 0.0 | 2.4 | 2.4 | 2.0 |  | 0.60 | 0.61 | 0.50 |
| 8 | *Botrylloides perspicuus* | H | N | 24.0 | 34.25 | M |  | 28.0 | 34.25 | M | 4.0 | 2.3 | 2.3 | 2.0 |  | 0.58 | 0.59 | 0.50 |
| 8 | *Botrylloides violaceus* | H | N | 31.0 | 34.25 | M |  | 39.0 | 34.25 | H | 8.0 | 2.6 | 2.7 | 1.8 |  | 0.65 | 0.67 | 0.46 |
| 8 | *Ciona intestinalis* | E | Y | 34.0 | 34.25 | M |  | 34.0 | 34.25 | M | 0.0 | 2.7 | 2.7 | 2.0 |  | 0.66 | 0.68 | 0.50 |
| 8 | *Diplosoma listerianum* | E | Y | 25.0 | 34.25 | M |  | 15.0 | 34.25 | M | −10.0 | 2.7 | 2.7 | 2.5 |  | 0.68 | 0.68 | 0.63 |
| 8 | *Microcosmus squamiger* | E | Y | 13.5 | 34.25 | M |  | 1.5 | 34.25 | M | −12.0 | 2.6 | 2.6 | 2.7 |  | 0.65 | 0.65 | 0.67 |
| 8 | *Polyandrocarpa zorritensis* | H | Y | 21.5 | 34.25 | M |  | 17.5 | 34.25 | M | −4.0 | 2.5 | 2.6 | 1.5 |  | 0.63 | 0.66 | 0.38 |
| 8 | *Polyclinum constellatum* | E | N | 34.5 | 34.25 | H |  | 34.5 | 34.25 | H | 0.0 | 2.5 | 2.6 | 2.0 |  | 0.64 | 0.65 | 0.50 |
| 8 | *Styela clava* | H | Y | 28.5 | 34.25 | M |  | 16.5 | 34.25 | M | −12.0 | 3.0 | 3.1 | 2.3 |  | 0.75 | 0.77 | 0.58 |
| 8 | *Styela plicata* | E | Y | 33.5 | 34.25 | M |  | 29.5 | 34.25 | M | −4.0 | 3.1 | 3.3 | 1.5 |  | 0.77 | 0.82 | 0.38 |
| 8 | *Symplegma brakenhielmi* | E | Y | 31.0 | 34.25 | M |  | 31.0 | 34.25 | M | 0.0 | 2.2 | 2.2 | 2.0 |  | 0.55 | 0.56 | 0.50 |
| 11 | *Amphibalanus subalbidus* | E | N | 32.0 | 26.25 | H |  | 24.0 | 20.50 | H | −8.0 | 2.7 | 2.7 | 2.3 |  | 0.66 | 0.67 | 0.58 |
| 11 | *Cordylophora caspia* | E | Y | 39.5 | 26.25 | H |  | 41.5 | 20.50 | H | 2.0 | 2.9 | 2.9 | 2.2 |  | 0.71 | 0.73 | 0.54 |
| 11 | *Ficopomatus enigmaticus* | H | Y | 38.0 | 26.25 | H |  | 42.0 | 20.50 | H | 4.0 | 3.1 | 3.1 | 2.8 |  | 0.77 | 0.78 | 0.71 |
| 11 | *Limnoperna fortunei* | H | Y | 20.0 | 26.25 | M |  | 10.0 | 20.50 | M | −10.0 | 2.9 | 2.8 | 3.7 |  | 0.73 | 0.70 | 0.92 |
| 12 | *Acartia (Acanthacartia) tonsa* | H | Y | 18.5 | 26.25 | M |  | 24.5 | 20.50 | H | 6.0 | 2.4 | 2.5 | 1.8 |  | 0.61 | 0.63 | 0.46 |
| 12 | *Alitta succinea* | H | Y | 24.0 | 26.25 | M |  | 30.0 | 20.50 | H | 6.0 | 2.9 | 2.8 | 3.2 |  | 0.71 | 0.70 | 0.79 |
| 12 | *Amphibalanus amphitrite* | E | N | 35.5 | 26.25 | H |  | 43.5 | 20.50 | H | 8.0 | 2.4 | 2.5 | 1.2 |  | 0.59 | 0.63 | 0.29 |
| 12 | *Amphibalanus improvisus* | E | N | 25.5 | 26.25 | M |  | 17.5 | 20.50 | M | −8.0 | 3.4 | 3.5 | 2.5 |  | 0.85 | 0.87 | 0.63 |
| 12 | *Amphibalanus venustus* | E | N | 34.5 | 26.25 | H |  | 34.5 | 20.50 | H | 0.0 | 2.3 | 2.4 | 1.0 |  | 0.56 | 0.60 | 0.25 |
| 12 | *Arcuatula senhousia* | H | Y | 21.5 | 26.25 | M |  | 11.5 | 20.50 | M | −10.0 | 2.9 | 2.9 | 3.0 |  | 0.74 | 0.73 | 0.75 |
| 12 | *Austrominius modestus* | H | Y | 40.0 | 26.25 | H |  | 40.0 | 20.50 | H | 0.0 | 3.3 | 3.3 | 3.2 |  | 0.83 | 0.83 | 0.79 |
| 12 | *Beroe ovata* | H | N | 26.0 | 26.25 | M |  | 14.0 | 20.50 | M | −12.0 | 2.9 | 2.8 | 3.0 |  | 0.71 | 0.71 | 0.75 |
| 12 | *Brachidontes pharaonis* | H | Y | 36.0 | 26.25 | H |  | 48.0 | 20.50 | H | 12.0 | 2.7 | 2.7 | 3.2 |  | 0.69 | 0.67 | 0.79 |
| 12 | *Bugula neritina* | E | Y | 34.0 | 26.25 | H |  | 44.0 | 20.50 | H | 10.0 | 3.0 | 3.1 | 2.2 |  | 0.74 | 0.77 | 0.54 |
| 12 | *Bugulina stolonifera* | E | N | 20.0 | 26.25 | M |  | 20.0 | 20.50 | M | 0.0 | 2.8 | 2.9 | 2.0 |  | 0.69 | 0.71 | 0.50 |
| 12 | *Carcinus maenas* | H | Y | 49.0 | 26.25 | H |  | 61.0 | 20.50 | H | 12.0 | 2.7 | 2.7 | 2.5 |  | 0.67 | 0.67 | 0.63 |
| 12 | *Carijoa riisei* | H | Y | 18.5 | 26.25 | M |  | 26.5 | 20.50 | H | 8.0 | 3.0 | 3.0 | 3.0 |  | 0.75 | 0.75 | 0.75 |
| 12 | *Cassiopea andromeda* | E | Y | 45.0 | 26.25 | H |  | 55.0 | 20.50 | H | 10.0 | 2.7 | 2.8 | 2.0 |  | 0.67 | 0.69 | 0.50 |
| 12 | *Cercopagis pengoi* | H | Y | 44.0 | 26.25 | H |  | 34.0 | 20.50 | H | −10.0 | 3.1 | 3.0 | 3.8 |  | 0.77 | 0.74 | 0.96 |
| 12 | *Charybdis (Charybdis) japonica* | H | Y | 39.0 | 26.25 | H |  | 31.0 | 20.50 | H | −8.0 | 2.6 | 2.6 | 2.3 |  | 0.65 | 0.65 | 0.58 |
| 12 | *Crassostrea virginica* | H | N | 22.0 | 26.25 | M |  | 16.0 | 20.50 | M | −6.0 | 2.9 | 3.0 | 2.7 |  | 0.73 | 0.74 | 0.67 |
| 12 | *Crepidula fornicata* | H | Y | 38.0 | 26.25 | H |  | 30.0 | 20.50 | H | −8.0 | 3.3 | 3.3 | 3.2 |  | 0.81 | 0.82 | 0.79 |
| 12 | *Doto kya* | E | Y | 6.0 | 26.25 | M |  | −4.0 | 20.50 | L | −10.0 | 2.2 | 2.1 | 2.7 |  | 0.54 | 0.53 | 0.67 |
| 12 | *Ectopleura crocea* | H | Y | 29.0 | 26.25 | H |  | 21.0 | 20.50 | H | −8.0 | 2.7 | 2.7 | 3.0 |  | 0.67 | 0.66 | 0.75 |
| 12 | *Eriocheir hepuensis* | E | Y | 30.5 | 26.25 | H |  | 36.5 | 20.50 | H | 6.0 | 3.0 | 3.2 | 2.0 |  | 0.76 | 0.79 | 0.50 |
| 12 | *Eriocheir sinensis* | H | Y | 42.0 | 26.25 | H |  | 32.0 | 20.50 | H | −10.0 | 3.1 | 3.2 | 2.0 |  | 0.78 | 0.81 | 0.50 |
| 12 | *Eualetes tulipa* | H | Y | 11.0 | 26.25 | M |  | 9.0 | 20.50 | M | −2.0 | 2.4 | 2.3 | 2.7 |  | 0.59 | 0.58 | 0.67 |
| 12 | *Gonionemus vertens* | H | Y | 9.0 | 26.25 | M |  | 1.0 | 20.50 | M | −8.0 | 2.8 | 2.7 | 3.3 |  | 0.69 | 0.67 | 0.83 |
| 12 | *Hediste diversicolor* | H | N | 15.0 | 26.25 | M |  | 15.0 | 20.50 | M | 0.0 | 2.3 | 2.4 | 2.0 |  | 0.58 | 0.59 | 0.50 |
| 12 | *Hemigrapsus sanguineus* | H | Y | 31.0 | 26.25 | H |  | 23.0 | 20.50 | H | −8.0 | 2.7 | 2.6 | 3.2 |  | 0.67 | 0.65 | 0.79 |
| 12 | *Hemigrapsus takanoi* | H | Y | 22.0 | 26.25 | M |  | 14.0 | 20.50 | M | −8.0 | 2.7 | 2.6 | 3.3 |  | 0.68 | 0.66 | 0.83 |
| 12 | *Leostyletus misakiensis* | E | N | 11.0 | 26.25 | M |  | 13.0 | 20.50 | M | 2.0 | 2.6 | 2.7 | 2.0 |  | 0.65 | 0.67 | 0.50 |
| 12 | *Macrobrachium sintangense* | E | Y | 33.0 | 26.25 | H |  | 23.0 | 20.50 | H | −10.0 | 2.9 | 3.0 | 2.0 |  | 0.73 | 0.76 | 0.50 |
| 12 | *Marenzelleria neglecta* | H | Y | 14.0 | 26.25 | M |  | 6.0 | 20.50 | M | −8.0 | 3.0 | 2.9 | 3.8 |  | 0.76 | 0.73 | 0.96 |
| 12 | *Megabalanus coccopoma* | E | Y | 44.0 | 26.25 | H |  | 56.0 | 20.50 | H | 12.0 | 2.4 | 2.5 | 1.7 |  | 0.60 | 0.62 | 0.42 |
| 12 | *Microchlamylla amabilis* | E | N | 9.5 | 26.25 | M |  | −0.5 | 20.50 | L | −10.0 | 2.5 | 2.5 | 3.0 |  | 0.64 | 0.62 | 0.75 |
| 12 | *Mnemiopsis leidyi* | H | Y | 42.0 | 26.25 | H |  | 42.0 | 20.50 | H | 0.0 | 3.0 | 3.0 | 2.7 |  | 0.74 | 0.75 | 0.67 |
| 12 | *Mycale (Mycale) grandis* | H | Y | 25.5 | 26.25 | M |  | 33.5 | 20.50 | H | 8.0 | 2.5 | 2.5 | 2.0 |  | 0.62 | 0.63 | 0.50 |
| 12 | *Mytilopsis sallei* | H | Y | 28.0 | 26.25 | H |  | 34.0 | 20.50 | H | 6.0 | 2.8 | 2.8 | 2.8 |  | 0.70 | 0.70 | 0.71 |
| 12 | *Mytilus galloprovincialis* | H | Y | 36.0 | 26.25 | H |  | 30.0 | 20.50 | H | −6.0 | 2.8 | 2.8 | 2.7 |  | 0.70 | 0.70 | 0.67 |
| 12 | *Palaemon macrodactylus* | H | Y | 19.0 | 26.25 | M |  | 11.0 | 20.50 | M | −8.0 | 2.9 | 2.7 | 3.8 |  | 0.71 | 0.68 | 0.96 |
| 12 | *Penaeus japonicus* | H | N | 24.0 | 26.25 | M |  | 20.0 | 20.50 | M | −4.0 | 2.9 | 2.8 | 3.3 |  | 0.72 | 0.71 | 0.83 |
| 12 | *Penaeus vannamei* | H | N | 30.0 | 26.25 | H |  | 24.0 | 20.50 | H | −6.0 | 2.8 | 2.7 | 3.2 |  | 0.69 | 0.68 | 0.79 |
| 12 | *Percnon gibbesi* | H | Y | 6.0 | 26.25 | M |  | 0.0 | 20.50 | L | −6.0 | 3.2 | 3.1 | 3.5 |  | 0.79 | 0.78 | 0.88 |
| 12 | *Perna perna* | H | Y | 23.0 | 26.25 | M |  | 29.0 | 20.50 | H | 6.0 | 2.7 | 2.7 | 2.7 |  | 0.68 | 0.68 | 0.67 |
| 12 | *Petricolaria pholadiformis* | H | Y | 14.0 | 26.25 | M |  | 6.0 | 20.50 | M | −8.0 | 3.0 | 2.9 | 3.5 |  | 0.75 | 0.73 | 0.88 |
| 12 | *Phyllorhiza punctata* | H | Y | 31.0 | 26.25 | H |  | 23.0 | 20.50 | H | −8.0 | 2.7 | 2.6 | 3.3 |  | 0.67 | 0.65 | 0.83 |
| 12 | *Pileolaria berkeleyana* | H | N | 3.0 | 26.25 | M |  | 3.0 | 20.50 | M | 0.0 | 2.5 | 2.6 | 1.5 |  | 0.62 | 0.65 | 0.38 |
| 12 | *Platorchestia platensis* | E | Y | 31.5 | 26.25 | H |  | 37.5 | 20.50 | H | 6.0 | 2.4 | 2.5 | 1.7 |  | 0.60 | 0.62 | 0.42 |
| 12 | *Potamocorbula amurensis* | H | Y | 24.0 | 26.25 | M |  | 16.0 | 20.50 | M | −8.0 | 2.9 | 2.8 | 3.7 |  | 0.72 | 0.70 | 0.92 |
| 12 | *Potamopyrgus antipodarum* | H | Y | 36.0 | 26.25 | H |  | 28.0 | 20.50 | H | −8.0 | 2.8 | 2.7 | 3.5 |  | 0.70 | 0.68 | 0.88 |
| 12 | *Pseudodiaptomus ardjuna* | E | N | 13.0 | 26.25 | M |  | 13.0 | 20.50 | M | 0.0 | 2.2 | 2.2 | 2.0 |  | 0.55 | 0.56 | 0.50 |
| 12 | *Pseudodiaptomus marinus* | H | Y | 13.0 | 26.25 | M |  | 3.0 | 20.50 | M | −10.0 | 2.9 | 2.9 | 3.0 |  | 0.73 | 0.73 | 0.75 |
| 12 | *Rapana venosa* | H | Y | 40.0 | 26.25 | H |  | 32.0 | 20.50 | H | −8.0 | 2.8 | 2.7 | 3.2 |  | 0.69 | 0.68 | 0.79 |
| 12 | *Rhithropanopeus harrisii* | H | Y | 27.0 | 26.25 | H |  | 21.0 | 20.50 | H | −6.0 | 2.8 | 2.8 | 3.2 |  | 0.70 | 0.69 | 0.79 |
| 12 | *Rhopalophthalmus tattersallae* | E | N | 1.0 | 26.25 | M |  | −9.0 | 20.50 | L | −10.0 | 2.6 | 2.7 | 2.0 |  | 0.65 | 0.67 | 0.50 |
| 12 | *Rhopilema nomadica* | H | Y | 29.0 | 26.25 | H |  | 29.0 | 20.50 | H | 0.0 | 2.7 | 2.8 | 1.7 |  | 0.66 | 0.69 | 0.42 |
| 12 | *Ruditapes philippinarum* | H | Y | 26.5 | 26.25 | H |  | 22.5 | 20.50 | H | −4.0 | 3.2 | 3.2 | 3.0 |  | 0.80 | 0.81 | 0.75 |
| 12 | *Schizoporella errata* | E | Y | 31.0 | 26.25 | H |  | 33.0 | 20.50 | H | 2.0 | 2.7 | 2.7 | 2.0 |  | 0.66 | 0.68 | 0.50 |
| 12 | *Tricellaria inopinata* | H | Y | 23.5 | 26.25 | M |  | 23.5 | 20.50 | H | 0.0 | 2.5 | 2.5 | 2.0 |  | 0.62 | 0.63 | 0.50 |
| 12 | *Trinchesia albocrusta* | E | N | 6.0 | 26.25 | M |  | −4.0 | 20.50 | L | −10.0 | 2.1 | 2.0 | 3.0 |  | 0.54 | 0.51 | 0.75 |
| 12 | *Tubastraea tagusensis* | E | Y | 29.0 | 26.25 | H |  | 29.0 | 20.50 | H | 0.0 | 2.7 | 2.8 | 2.0 |  | 0.67 | 0.69 | 0.50 |
| 12 | *Watersipora subtorquata* | H | Y | 29.0 | 26.25 | H |  | 29.0 | 20.50 | H | 0.0 | 2.4 | 2.4 | 2.0 |  | 0.60 | 0.61 | 0.50 |
| 18 | *Caulerpa cylindracea* | H | Y | 42.0 | 27.50 | H |  | 32.0 | 28.25 | H | −10.0 | 2.2 | 2.3 | 1.7 |  | 0.56 | 0.58 | 0.42 |
| 18 | *Caulerpa lamourouxii* | E | N | 39.0 | 27.50 | H |  | 35.0 | 28.25 | H | −4.0 | 2.6 | 2.7 | 2.2 |  | 0.65 | 0.66 | 0.54 |
| 18 | *Caulerpa taxifolia* | H | Y | 42.0 | 27.50 | H |  | 32.0 | 28.25 | H | −10.0 | 2.3 | 2.4 | 1.2 |  | 0.57 | 0.60 | 0.29 |
| 18 | *Codium fragile fragile* | H | Y | 28.5 | 27.50 | H |  | 18.5 | 28.25 | M | −10.0 | 2.0 | 2.2 | 1.0 |  | 0.51 | 0.54 | 0.25 |
| 18 | *Eucheuma* spp. | H | Y | 26.5 | 27.50 | M |  | 28.5 | 28.25 | H | 2.0 | 2.6 | 2.7 | 2.2 |  | 0.65 | 0.67 | 0.54 |
| 18 | *Gracilaria tikvahiae* | H | N | 25.5 | 27.50 | M |  | 21.5 | 28.25 | M | −4.0 | 2.5 | 2.6 | 2.0 |  | 0.62 | 0.64 | 0.50 |
| 18 | *Grateloupia filicina* | E | N | 26.5 | 27.50 | M |  | 38.5 | 28.25 | H | 12.0 | 2.7 | 2.8 | 2.0 |  | 0.67 | 0.69 | 0.50 |
| 18 | *Grateloupia turuturu* | H | Y | 25.5 | 27.50 | M |  | 17.5 | 28.25 | M | −8.0 | 2.7 | 2.8 | 2.2 |  | 0.67 | 0.69 | 0.54 |
| 18 | *Hypnea musciformis* | E | Y | 48.0 | 27.50 | H |  | 54.0 | 28.25 | H | 6.0 | 2.7 | 2.7 | 2.2 |  | 0.67 | 0.68 | 0.54 |
| 18 | *Polysiphonia brodiei* | E | Y | 30.0 | 27.50 | H |  | 30.0 | 28.25 | H | 0.0 | 3.2 | 3.2 | 3.2 |  | 0.80 | 0.80 | 0.79 |
| 18 | *Ulva australis* | H | N | 26.5 | 27.50 | M |  | 26.5 | 28.25 | M | 0.0 | 1.8 | 1.9 | 1.0 |  | 0.45 | 0.47 | 0.25 |
| 18 | *Ulva ohnoi* | E | N | 24.5 | 27.50 | M |  | 24.5 | 28.25 | M | 0.0 | 1.6 | 1.7 | 1.0 |  | 0.40 | 0.42 | 0.25 |
| 21 | *Alexandrium minutum* | E | Y | 44.0 | 28.50 | H |  | 56.0 | 28.25 | H | 12.0 | 3.1 | 3.2 | 2.2 |  | 0.78 | 0.81 | 0.54 |
| 21 | *Dinophysis caudata* | E | N | 35.5 | 28.50 | H |  | 47.5 | 28.25 | H | 12.0 | 3.2 | 3.2 | 2.8 |  | 0.79 | 0.80 | 0.71 |
| 21 | *Gymnodinium catenatum* | E | Y | 44.0 | 28.50 | H |  | 54.0 | 28.25 | H | 10.0 | 3.1 | 3.2 | 2.2 |  | 0.77 | 0.80 | 0.54 |
| 21 | *Heterosigma akashiwo* | E | N | 44.0 | 28.50 | H |  | 56.0 | 28.25 | H | 12.0 | 3.0 | 3.1 | 2.2 |  | 0.75 | 0.78 | 0.54 |
| 21 | *Karenia mikimotoi* | E | Y | 32.0 | 28.50 | H |  | 44.0 | 28.25 | H | 12.0 | 3.2 | 3.3 | 2.8 |  | 0.80 | 0.82 | 0.71 |
| 21 | *Karenia selliformis* | E | N | 43.0 | 28.50 | H |  | 55.0 | 28.25 | H | 12.0 | 3.1 | 3.2 | 2.2 |  | 0.77 | 0.80 | 0.54 |
| 21 | *Kryptoperidinium foliaceum* | E | Y | 42.0 | 28.50 | H |  | 54.0 | 28.25 | H | 12.0 | 3.0 | 3.1 | 2.2 |  | 0.75 | 0.77 | 0.54 |
| 21 | *Margalefidinium polykrikoides* | E | Y | 44.0 | 28.50 | H |  | 56.0 | 28.25 | H | 12.0 | 3.1 | 3.2 | 2.2 |  | 0.77 | 0.80 | 0.54 |
| 21 | *Myrionema orbiculare* | E | N | 6.0 | 28.50 | M |  | 8.0 | 28.25 | M | 2.0 | 2.5 | 2.5 | 2.0 |  | 0.61 | 0.63 | 0.50 |
| 21 | *Prorocentrum mexicanum* | E | N | 25.0 | 28.50 | M |  | 21.0 | 28.25 | M | −4.0 | 2.5 | 2.6 | 2.0 |  | 0.63 | 0.64 | 0.50 |
| 21 | *Prorocentrum micans* | E | N | 34.5 | 28.50 | H |  | 46.5 | 28.25 | H | 12.0 | 2.7 | 2.7 | 2.5 |  | 0.67 | 0.67 | 0.63 |
| 21 | *Prymnesium parvum* | H | Y | 36.5 | 28.50 | H |  | 36.5 | 28.25 | H | 0.0 | 1.9 | 1.9 | 1.8 |  | 0.48 | 0.48 | 0.46 |
| 21 | *Pyrodinium bahamense* | E | N | 41.0 | 28.50 | H |  | 47.0 | 28.25 | H | 6.0 | 3.1 | 3.2 | 2.5 |  | 0.78 | 0.80 | 0.63 |
| 21 | *Sargassum fluitans* | E | Y | 33.0 | 28.50 | H |  | 39.0 | 28.25 | H | 6.0 | 2.9 | 3.1 | 2.0 |  | 0.74 | 0.77 | 0.50 |
| 21 | *Sargassum muticum* | H | Y | 33.5 | 28.50 | H |  | 23.5 | 28.25 | M | −10.0 | 2.9 | 2.9 | 2.5 |  | 0.72 | 0.73 | 0.63 |
| 21 | *Tintinnopsis ampla* | E | N | 25.0 | 28.50 | M |  | 37.0 | 28.25 | H | 12.0 | 2.8 | 3.0 | 1.3 |  | 0.70 | 0.74 | 0.33 |
| 21 | *Undaria pinnatifida* | H | Y | 37.0 | 28.50 | H |  | 33.0 | 28.25 | H | −4.0 | 2.7 | 2.8 | 1.5 |  | 0.67 | 0.70 | 0.38 |

**Reference list for references in Table S1**

Aein-Jamshid, K., Fallahi, M., Mohseni-Zadeh, F., Izad-Panahi, G., Tavakoli, H., ... & Esmaeili, A. R., (2014). *A survey on the effect of Cochlodinium. sp bloom on shrimp culture complexes and hatcheries activities in Bushehr Province*. Iranian Fisheries Research Organization. Available from: <http://aquaticcommons.org/25457/1/43549.pdf>

Al-Azri, A. R., Al-Hashmi, K. A., Al-Habsi, H., Al-Azri, N., & Al-Khusaibi, S., (2015). Abundance of harmful algal blooms in the coastal waters of Oman: 2006–2011. *Aquatic Ecosystem Health & Management, 18(3),* 269–281.

Al-Faisal, A. J. M., Mutlak, F. M., & Abdullah, S. A., (2014). Exotic freshwater fishes in the Southern Iraq. *Marsh Bulletin*, *9(1*), 65-78.

Al-Faisal, A. J., & Mutlak, F. M., (2014). First record of the Nile tilapia *Oreochromis niloticus* (Linnaeus, 1758), from the Shatt Al-Arab River, Southern Iraq. *Mesopotamian Journal of Marine Science*, *29(1*), 45-50.

Al-Hassan, L. A. J., & Miller, P. J., (1987). *Rhinogobius brunneus* (Gobiidae) in the Arabian Gulf. *Japanese Journal of Ichthyology*, *33(4),* 405-408.

Al-Kahem, H. F., Al-Ghanim, A. A., & Ahmad, Z., (2007). Studies on feeding ecology of sailfin molly (*Poecilia latipinna*) dwelling in Wadi Haneefah stream, Riyadh. *Pakistan Journal of Biological Sciences*, *10(2*), 335-341.

Al-Yamani, F. Y., Skryabin, V. & Durvasula, S. R. V., (2015). Suspected ballast water introductions in the Arabian Gulf. *Aquatic Ecosystem Health & Management, 18(3),* 282–289.

Al-Yamani, F. Y., Skryabin, V., Boltachova, N., Revkov, N., Makarov, M., … & Kolesnikova, E., (2014). *Illustrated atlas on the zoobenthos of Kuwait.* Produced for the Kuwait Institute for Scientific Research: Safat.

Al-Yamani, F. Y., Saburova, M., & Polikarpov, I., (2012). A preliminary assessment of harmful algal blooms in Kuwait's marine environment, Aquatic Ecosystem Health & Management, *15(1*), 64-72.

Arndt, E. A., (1989). Ecological, physiological and historical aspects of brackish water fauna distribution. Reproduction, genetics and distribution of marine organisms. *Olsen & Olsen, Fredensborg*, 327-338.

Bartley D. M., (2006). *Introduced species in fisheries and aquaculture: information for responsible use and control.* Rome, Italy, FAO: unpaginated.

Bohm, A., (1931). Peridineen aus dem Persischen Golf und dem Golf von Oman. *Archiv fur Protistenkunde*, *74*, 188-197

Bollens, S. M., Cordell, J. R., Avent, S. & Hooff, R., (2002). Zooplankton invasions: a brief review, plus two case studies from the northeast Pacific Ocean. *Hydrobiologia,* *480(1–3*), 87–110.

Canonico, G., Arthington, A., McCarary, J. K., and Thieme, M. L., (2005). The effects of introduced tilapia’s on biodiversity. *Aquatic Conservation, Marine and Freshwater Ecosystems, 15*, 463–488.

Carlton, J. T. & Geller, J. B., (1993). Ecological roulette: the global transport of nonindigenous marine organisms. *Science, 261(5117),* 78–82.

Clark, P. F., Abdul-Sahib, I. M., & Al-Asadi, M. S. (2006). The first record of *Eriocheir sinensis* H. Milne Edwards, 1853 (Crustacea: Brachyura: Varunidae) from the Basrah area of southern Iraq. *Aquatic Invasions, 1(2),* 51–54.

Coad B.W. (1995). Freshwater fishes of Iran. *Acta Scientiarum Naturalium Academiae Scientiarum Bohemoslovacae Brno, 29(1)*, 1–64.

Creed, J. C., Fenner, D., Sammarco, P., Cairns, S., Capel, K., … & Oigman-Pszczol, S., (2017). The invasion of the azooxanthellate coral *Tubastraea* (Scleractinia: Dendrophylliidae) throughout the world: history, pathways and vectors. *Biological invasions*, *19(1),* 283-305.

Cruz, E. M., Ridha, M., and Abdullah, M. S., (1990). Production of the African freshwater tilapia *Oreochromis spilurus* (Günther) in seawater. *Aquaculture, 81(1),* 41–48

Dobretsov, S. (2015). Biofouling on artificial substrata in Muscat waters. *Journal of Agricultural and Marine Sciences, 20*, 24–29.

Dore & Frimodt, (1987). *An Illustrated Guide to Shrimp of the World*. An Osprey Book. Published by Van Nostrand Reinhold New York. 229pp

El-Sayed, A. F. M., (2006). Tilapia culture in saltwater: environmental requirements, nutritional implications and economic potentials. In *Eighth Symposium on Advances in Nutritional Aquaculture.* November (pp. 15-17).

Engesmo, A., Eikrem, W., Seoane, S., Smith, K., Edvardsen, B., …& Tomas, C.R., (2016). New insights into the morphology and phylogeny of *Heterosigma akashiwo* (Raphidophyceae), with the description of *Heterosigma minor* sp. nov. *Phycologia, 55(3*), 279–294.

Fotonov, P. W., Ruiz, G. M., Steves, B., & Carlton, J. T. (2014). National Exotic Marine and Estuarine Species Information System (NEMESIS). Available online at: <http://invasions.si.edu/nemesis>

Froese, R., & Pauly, D., (2004). FishBase DVD. Penang, Malaysia: Worldfish Centre. Available online at [www.fishbase.org](http://www.fishbase.org)

Glibert, P. M., Landsberg, J. H., Evans, J. J., Al-Sarawi, M. A., Muna Faraj, …& Shoemaker, C., (2002). A fish kill of massive proportion in Kuwait Bay, Arabian Gulf, 2001: the roles of bacterial disease, harmful algae, and eutrophication. *Harmful Algae,* *1(2),* 215–231.

González-Félix, M. L., Gatlin, D. M., Perez-Velazquez, M., Webb, K., García-Ortega, A., & Hume, M., (2018). Red drum *Sciaenops ocellatus* growth and expression of bile salt-dependent lipase in response to increasing dietary lipid supplementation. *Fish Physiology and Biochemistry*, 1-13.

Grabe, S. A., (1989). Some aspects of the biology of *Rhopalophthalmus tattersallae* Pillai, 1961 (Crustacea, Mysidacea) and extension of range into the khor al sabiya, Kuwait (Arabian Gulf). *Proceedings of the Biological Society of Washington,* *102(3*), 726–731.

Hallegraeff, G. M., (2015). Transport of harmful marine microalgae *via* ship's ballast water: Management and mitigation with special reference to the Arabian Gulf region. *Aquatic Ecosystem Health & Management, 18(3),* 290–298.

Hallegraeff, G. M., (1995). *Harmful algal blooms: A global overview.* *In:* Hallegraeff, G.M., D.M. Anderson & A.D. Cembella (eds.), Manual on Harmful Marine Microalgae, IOC Manuals and Guides No. 33, UNESCO, Paris: 1–22

Hallegraeff, G. M. & Bolch, C. J., (1992). Transport of dinoflagellate cysts in ship's ballast water: Implications for plankton biogeography and aquaculture. *Journal of Plankton Research, 14,* 1067–1084.

Hashim, A. A., (2010) Occurrence of the Chinese mitten crab *Eriocheir sinensis* (H. Milne Edwards) in South Iraq. *Mesopotamian Journal of Marine Science, 25*, 31–36.

Heil, C. A., Glibert, P. M., Al-Sarawi, M. A., Faraj, M., Behbehani, M. & Husain, M., (2001). First record of a fish-killing *Gymnodinium* sp. bloom in Kuwait Bay, Arabian Sea: chronology and potential causes. *Marine Ecology Progress Series, 214*, 15–23.

Holthuis, L. B., & Hassan, A. M. (1975). The introduction of *Palaemon elegans* Rathke, 1837 (Decapoda, Natantia) in Lake Abu-Dibic, Iraq. *Crustaceana,* 141–148.

Ibrahim, F. H., & Al-Shawi, I. J. M., (2015). Potential Harmful Dinoflagellates of Iraqi Coastal Marine Waters. *International Journal of Marine Science*, *5*.

IUCN, (2016). IUCN SSC Invasive Species Specialist Group, *Workshop outputs - Ministry of Climate Change and Environment (MoCCE), United Arab Emirates and International Union for Conservation of Nature (IUCN) – Validation and Prioritization Workshop*. Struik-Random House Publishers. Cape Town.

John, D. M. & Al-Thani, R. F., (2014). Benthic marine algae of the Arabian Gulf: a critical review and analysis of distribution and diversity patterns. *Nova Hedwigia*, 98, 341–392.

Jonassen, T. M., Pittman, K., & Imsland, A. K., (1997). Seawater acclimation of tilapia, *Oreochromis spilurus spilurus* Günter, fry and fingerlings. *Aquaculture Research*, *28(3),* 205-214.

Jones, D. A., (1986). *A field guide to the sea shores of Kuwait and the Arabian Gulf*. University of Kuwait, Kuwait.

Khaefi, R., Esmaeili, H. R., Zareian, H., & Babaei, S., (2014). The first record of the redbelly tilapia, *Tilapia zillii* (Gervais, 1848), in freshwaters of Iran. *Turkish Journal of Zoology*, *38(1),* 96-98.

Khaleghi, M., (2016). First record of Six Species of Ascidians (Tunicata: Ascidiacea) from Chabahar Bay (Gulf of Oman), Iran. *Journal of Applied Environmental Biological Science, 6(2),* 122-129.

Klein, J., & Verlaque, M., (2008). The *Caulerpa racemosa* invasion: a critical review. *Marine Pollution Bulletin*, *56(2),* 205-225.

Koutsikos, N., Vardakas, L., Kalogianni, E., & Economou, A. N., (2018). Global distribution and climatic match of a highly traded ornamental freshwater fish, the sailfin molly *Poecilia latipinna* (Lesueur, 1821). *Knowledge & Management of Aquatic Ecosystems*, *419*, 23.

Lin, C. K. & Suresh, A. V., (1992). Tilapia culture in saline waters: a review. *Aquaculture (Netherlands), 106(3–4),* 201–226.

McDonald, E. M., (1987). Interactions between a phytoplanktivorous fish, *Oreochromis aureus*, and two unialgal forage populations. *Environmental Biology of Fishes, 18*, 229–234.

Meliane, I., & Ramos-Espla, M. A. A., (2001). Records of Ascidians (Chordata, Tunicata) from Oman, South East of Arabian Peninsula. *Conference proceedings of 1^st^ International Conference on Fisheries, Aquaculture and the Environment in the North West Indian Ocean*. Sultan Qaboos University, Muscat, Sultanate of Oman, pp. 37–41.

Mohamed, H. H., (2011). First record of the marine calanoid copepod *Pseudodiaptomus cf ardjuna* from Shatt Al-Arab River, Iraq. *Mesopotamian Journal of Marine Science*, *26(1*), 59-68.

Mohebbi, G., Nabipour, I., Vazirizadeh, A., Vatanpour, H., Farrokhnia, M., … & Bargahi, A., (2018). Acetylcholinesterase inhibitory activity of a neurosteroidal alkaloid from the upside-down jellyfish *Cassiopea andromeda* venom. *Revista Brasileira de Farmacognosia*, *28(5),* 568-574.

Nabipour, I., Moradi, M., & Mohebbi, G. H., (2015). A first record on population of the alien venomous jellyfish, *Cassiopea andromeda* (Forsskål, 1775) (Cnidaria: Scyphozoa: Rhizostomea) in the Nayband Lagoon from Bushehr-Iran (Persian Gulf). *Journal of Chemical and Pharmaceutical Research*, *7(3),* 1710-1713.

Naderloo, R., (2014). Invasive Hepu mitten crab, *Eriocheir hepuensis* (Crustacea: Decapoda: Brachyura: Varunidae) from the Iranian marshland in the northern Persian Gulf estuarine system. *Marine Biodiversity Records, 7*, e23.

Naser, H. A., (2017). Variability of marine macrofouling assemblages in a marina and a mariculture centre in Bahrain, Arabian Gulf. *Regional Studies in Marine Science, 16*, 162–170

Naser, M.D., Rainbow, P.S., Clark, P.F., Yasser, A.G., and Jones, D.S., (2015). The barnacle *Amphibalanus improvisus* (Darwin, 1854), and the mitten crab *Eriocheir*: one invasive species getting off on another! *BioInvasions Records, 4*, 205–209.

Naser, M.D., Page, T.J., Ng, N.K., Apel, M., Yasser, A.G., Bishop, J.M., Ng, P.K. and Clark, P.F., (2012). Invasive records of *Eriocheir hepuensis* Dai, 1991 (Crustacea: Brachyura: Grapsoidea: Varunidae): Implications and taxonomic considerations. *BioInvasions Records, 1*, 186.

Nasrolahi A; Farahani F, Saifabadi SJ, (2006). Effect of salinity on larval development and survival of the Caspian Sea Barnacle, *Balanus improvisus* Darwin (1854). *Journal of Biological Science, 6(6)*, 1103–1107.

Norman-López, A., & Bjørndal, T**.,** (2009). Is tilapia the same product worldwide or are markets segmented? *Aquaculture Economics and Management,* *13(2),* 138–154.

Pagad, S., Hayes, K., Katsanevakis, S. & Costello, M. J., (2018) *World Register of Introduced Marine Species* (WRIMS). Available online at: <http://www.marinespecies.org/introduced>.

Pirian, K., Piri, K., Sohrabipour, J., Jahromi, S. T., & Blomster, J., (2016). Molecular and morphological characterisation of *Ulva chaugulii, U. paschima and U. ohnoi* (Ulvophyceae) from the Persian Gulf, Iran. *Botanica Marina*, *59(2-3),* 147-158.

Pullin, R. S., Palmares, M. L., Casal, C. V., Dey, M., M., & Pauly, D., (1997). Environmental impacts of tilapias. In: Fitzsimmons K, ed*. Proceedings of the 4th International Symposium on Tilapia in Aquaculture. Ithaca, NY, USA*: Northeast Regional Agricultural Engineering Service, 554–572.

Richlen, M. L., Morton, S. L., Jamali, E. A., Rajan, A., & Anderson, D. M., (2010). The catastrophic 2008–2009 red tide in the Arabian Gulf region, with observations on the identification and phylogeny of the fish-killing dinoflagellate *Cochlodinium polykrikoides*. *Harmful Algae, 9(2),* 163–172.

Rountos, K. J., Gobler, C. J. & Pikitch, E. K., (2017). Ontogenetic Differences in Swimming Behaviour of Fish Exposed to the Harmful Dinoflagellate *Cochlodinium polykrikoides. Transactions of the American Fisheries Society, 146,* 1081–1091.

Saad, G. A., (2016). A Coccidian Parasite Inhabiting the GI Tract and Leucocytes of *Styela plicata* (Lesuaer, 1823) and *Ciona intestinalis* (Linnaeus, 1767) Sampled from the Arabian Gulf (Saudi Arabia). *International Journal of Marine Science, 6(18*), 1-14.

Saburova, M., Polikarpov, I., & Al-Yamani, F., (2012). First record of *Kryptoperidinium foliaceum* (Dinophyceae: Peridiniales) from a hypersaline environment in Kuwait, north-western Arabian Gulf. *Marine Biodiversity Records, 5*, e104.

Shahdadi, A, Sari A, & Naderloo, R., (2014). A checklist of the barnacles (Crustacea: Cirripedia: Thoracica) of the Persian Gulf and Gulf of Oman with nine new records. *Zootaxa, 3784*, 201–223.

Shapoori, M., & Gholami, M., (2014). Effect of a ballast water treatment system on survivorship of natural populations of marine plankton in Persian Gulf, Iran. *Marine Science, 4(2),* 44–48.

Sherly, S. & Sambhu, C., (2016). Acclimation and tolerance of marine sabaki tilapia, *Oreochromis spilurus* (Günther) in fresh water. *Journal of Aquatic Biology & Fisheries*, *4*, 134-136.

Siddiqui, A. Q., & Al-Harbi, A. H., (1995). Evaluation of three species of tilapia, red tilapia and a hybrid tilapia as culture species in Saudi Arabia. *Aquaculture, 138(1/4),* 145–157.

Siddiqui, A. Q., Howlader, M. S., & Adam, A. B., (1989). Culture of Nile tilapia, *Oreochromis niloticus* (L.), at three stocking densities in outdoor concrete tanks using drainage water. *Aquaculture Research*, *20(1), 49-58.*

Silva, P. C., Basson, P. W. & Moe, R. L., (1996). Catalogue of the benthic marine algae of the Indian Ocean. *University of California Publications in Botany, 79,* 1–1259.

Singh, A., Hårding, K., Reddy, H. R. V. & Godhe, A., (2014). An assessment of *Dinophysis* blooms in the coastal Arabian Sea. *Harmful Algae*, *34*, 29-35.

Sohrabipour, J., & Rabii, R., (1999). A list of marine algae of seashores of Persian Gulf and Oman Sea in the Hormozgan Province. *Iranian Journal of Botany, 8(1),* 131–162.

Teimori, A., Motamedi, M., & Hesni, M. A., (2017). Translocation and new geographical distribution of the invasive Redbelly Tilapia, *Coptodon zillii* (Gervais, 1848) (Teleostei: Cichlidae) in southern Iran. *Check List,* *13(1*), 2051.

Uddin, S., Fowler, S. W., Behbehani, M., & Metian, M., (2017). 210Po bioaccumulation and trophic transfer in marine food chains in the northern Arabian Gulf. *Journal of Environmental Radioactivity, 174,* 23–29.

Usup, G., Ahmad, A., Matsuoka, K., Lim, P. T., & Leaw, C. P., (2012). Biology, ecology and bloom dynamics of the toxic marine dinoflagellate *Pyrodinium bahamense*. *Harmful Algae*, *14*, 301-312.

Valikhania, H., Abdoli, A., Kiabi, B. H., & Nejat, F., (2016). First record and distribution of the blue tilapia, Oreochromis aureus (Steindachner, 1864) (Perciformes: Cichlidae) in inland waters of Iran. *Iranian Journal of Ichthyology, 3(1),* 19–24.

Vanneyre, W. K. & John, D. M., (2014). Note on *Caulerpa racemosa* var*. lamourouxii* f. *requienii*: the first record of a potentially invasive non-native green seaweeds from the Arabian Gulf. *The Phycologist, 86.*

Victor, R., & Al-Makki, N., (2000). Escaped tilapia in an Arabian pond: a biological assessment. *Internationale Vereinigung für theoretische und angewandte Limnologie: Verhandlungen*, *27(2),* 762–767.

World Health Organization (WHO), (1997). *Opportunistic settlers and the problem of the ctenophore Mnemiopsis leidyi invasion in the Black Sea*. Report produced by Joint Group of Experts on the Scientific Aspects of Marine Environmental Protection. In: GESAMP reports and studies no. 58, 84pp.
